# iMOKA: *k*-mer based software to analyze large collections of sequencing data

**DOI:** 10.1186/s13059-020-02165-2

**Published:** 2020-10-13

**Authors:** Claudio Lorenzi, Sylvain Barriere, Jean-Philippe Villemin, Laureline Dejardin Bretones, Alban Mancheron, William Ritchie

**Affiliations:** 1 IGH, Centre National de la Recherche Scientifique, University of Montpellier, Montpellier, France; 2 LIRMM, Université de Montpellier, CNRS, Montpellier, France

**Keywords:** *k*-mer, NGS analysis, Personalized medicine, Bioinformatics software, Data reduction, Machine learning

## Abstract

iMOKA (interactive multi-objective *k*-mer analysis) is a software that enables comprehensive analysis of sequencing data from large cohorts to generate robust classification models or explore specific genetic elements associated with disease etiology. iMOKA uses a fast and accurate feature reduction step that combines a Naïve Bayes classifier augmented by an adaptive entropy filter and a graph-based filter to rapidly reduce the search space. By using a flexible file format and distributed indexing, iMOKA can easily integrate data from multiple experiments and also reduces disk space requirements and identifies changes in transcript levels and single nucleotide variants. iMOKA is available at https://github.com/RitchieLabIGH/iMOKA and Zenodo 10.5281/zenodo.4008947.

## Background

Studies of variation in gene expression have considerably advanced knowledge of disease etiology and classification [1–3]. To capitalize on genomic data generated from numerous clinical studies, recent initiatives have aggregated high-throughput sequencing (HTS) experiments from multiple cohorts that measure gene expression, RNA isoform usage, and genome variation. For example, the Genomic Data Commons program controls access to over 84,000 cases [4]. Still, despite these efforts to aggregate and provide data from multiple studies, their computational analysis and integration presents a major challenge; each type of HTS data requires specific bioinformatics pipelines that need to be implemented by a bioinformatics specialist. In addition, most of these approaches require reference genomes or transcriptomes and thus cannot inherently account for the diversity in non-reference transcripts or individual variations [5]. To alleviate the requirement of a reference, recent methodologies use *k-mer* representation; they directly compare the counts of nucleotide sequences of length *k* between samples [6]. These *k*-mer based approaches have been core to the field of metagenomics, where they are used to discover unique *k*-mers or *k*-mer signatures to classify organisms [7, 8]. However, when translated to mammalian genomes, *k*-mer representation results in a *k*-mer count matrix with as many columns as there are samples and as many rows as there are *k*-mers, generally billions. Exploring such large matrices to find biologically relevant *k*-mers is intractable unless the analysis focuses only on a very small subset of the sequencing data [5] or by using metaheuristics that provide partial solutions [9].

Here we present iMOKA (interactive multi-objective *k*-mer analysis), a novel approach and software that allows non-specialists to make use of *k*-mers to explore large amounts of mammalian sequencing data. This approach is agnostic of the type of sequencing data used, is not biased towards annotated genetic elements, and can analyze transcript levels and single nucleotide variations in one pass. Importantly, iMOKA is interactive; it allows the user to import and merge samples from different studies and tailor their exploration of *k*-mers to specific genomic elements of interest such as splicing events, mutations, or global gene expression. We tested iMOKA on four clinical datasets: the classification of breast cancer subtypes and response to chemotherapy of breast, ovarian cancer, and diffuse large B cell lymphoma (DLBCL). We find that iMOKA found features that are more accurate than classical bioinformatics approaches, takes up less space, uses less memory, has faster runtimes, and can be run on a computer cluster or on a laptop.

## Results

### iMOKA design

**Fig. 1:**
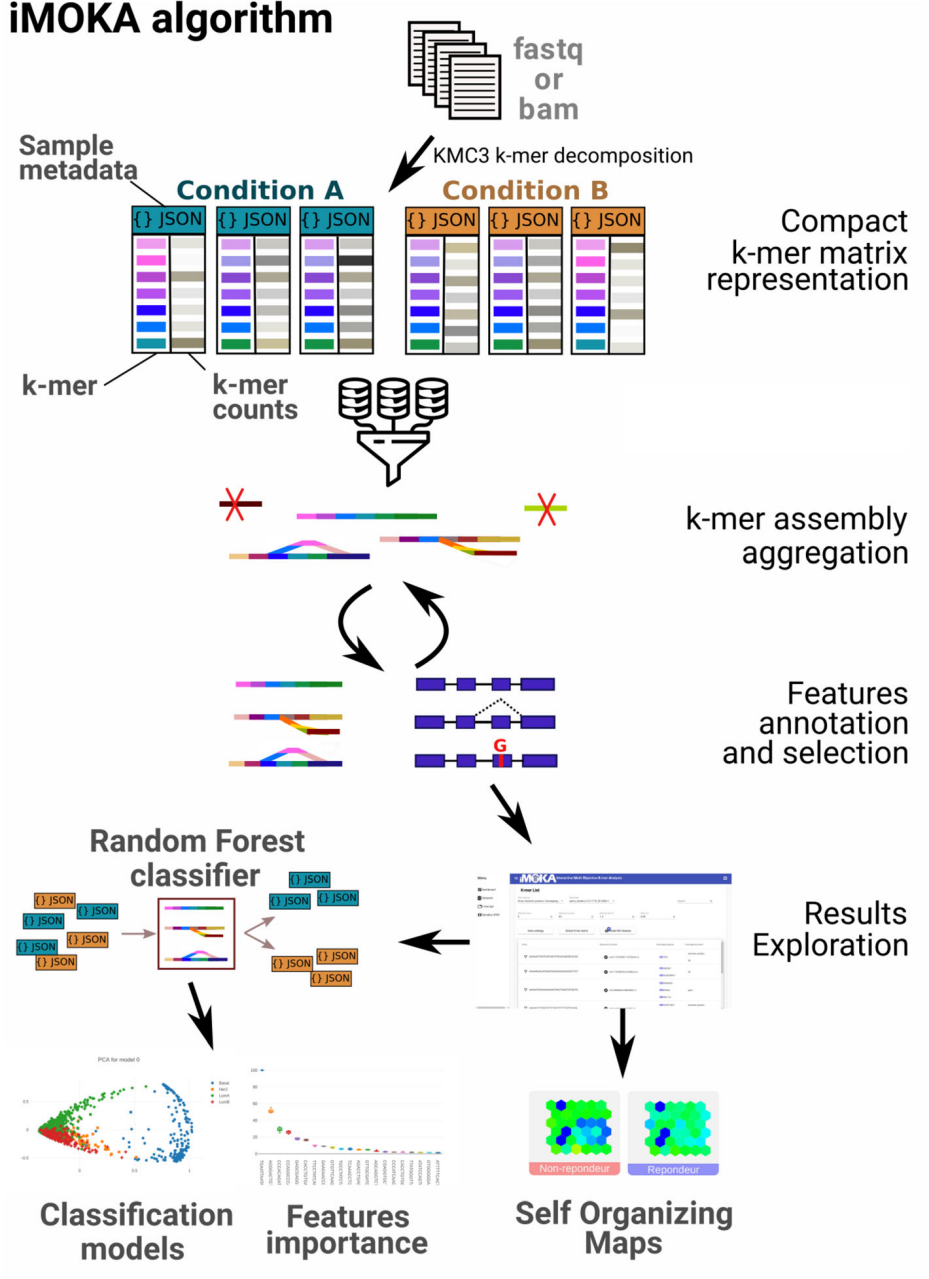
Overview of the iMOKA algorithm. The software accepts sequencing reads in FASTQ, FASTA, BAM formats, or SRR identifiers. The *k*-mer count in each file is calculated and stored using a dedicated file format. *k*-mers are then filtered using an Entropy boosted Bayes filter with Monte Carlo cross validation to obtain the *k*-mers that are able to classify the input samples. These are combined into graphs and annotated using GMAP or another user-defined aligner. The final list of highly informative *k*-mers can be explored using the graphical interface to create classification models, inspect individual *k*-mers, and detect sample outliers using self-organizing maps

iMOKA imports sequencing files in FASTQ, FASTA, BAM format, or SRR identifiers via its user interface. It then counts the occurrences of all sequences of given length *k* (default 31) [9] using the KMC3 software [10] in each sample (Fig. 1). It then extracts labels from the sequencing metadata so that the user can define groups they wish to compare. Importantly, each sample is stored as a sorted vector of *k*-mer counts in a dedicated binary file using a custom prefix-suffix structure that drastically reduces the disk space requirements (“Methods” section). For each sample, a JSON file is created that contains metadata and a rescaling factor for *k*-mer count normalization that allows the user to remove or add samples without having to recalculate an entire *k*-mer matrix. It then uses our feature reduction step that combines a Bayes classifier augmented by an adaptive entropy filter to rapidly remove non-relevant *k*-mers (Fig. S1). The aim of this filter is to evaluate each *k*-mer individually by combining the accuracy of the Bayes classifier with the speed of calculating Shannon’s entropy. This evaluation is performed using a Monte Carlo cross validation with a high number of iterations and an early break (“Methods” section) that efficiently reduces overfitting and generates predictions that overcome batch effects. In order to reduce the number of features evaluated, the entropy filter works simultaneously and, learning from the entropies of the *k*-mers that successfully passed the accuracy filter, discards *k*-mers with low entropy. Following this filtering, *k*-mers for which the sequences overlap are assembled into graph structures. These are used to aggregate the *k*-mers that are likely to have been generated from the same biological sequence and are used to eliminate false positive *k*-mers that are mainly singletons (1 *k*-mer) or very short branches in the graph structure. Bifurcations or bubbles in these graphs generally arise from the existence of multiple sequence isoforms that differ by point mutations or alternative splicing events [11]. By combining this graph assembly with the relatively permissive Bayesian filter, we are able to generate a list of informative *k*-mers in a manner that is fast and accurate.

iMOKA allows the user to align the *k*-mer graphs to a reference genome to annotate them with known genomic features such as known RNA transcripts, point mutations, or mRNA splicing events. iMOKA provides a random forest classifier that uses filtered *k*-mer graphs as features (Supplementary methods) and provides the user with a classification model and a sorted list of *k*-mer graphs that were most used in the tree models and that are thus of higher interest (Fig. 1). The user may even build classification models based solely on specific genomic features such as point mutations or gene expression for example. Finally, iMOKA uses self-organizing map clustering on the *k*-mer graphs to enable users to identify subgroups or outliers amongst their input samples.

### Benchmarking datasets and algorithms

iMOKA uses a *k*-mer based analysis to detect sequence features and create classification models from large cohorts of mammalian RNA sequencing data. To test its performance, we selected four studies that were distinct in their data structures, classification objectives, and sizes. The first was a non-binary classification of 1038 patients aiming to define 4 subtypes of breast cancer which were luminal A (LumA), luminal B (LumB), HER2-enriched (HER2), and basal-like. The second was a cohort of 240 ovarian cancer patients where the objective was to predict response to chemotherapy. The third was a smaller cohort of 118 breast cancer patients where the objective was also to predict response to chemotherapy. The last was an even smaller cohort of 17 DLBCL patients divided according to their responsiveness to the chemotherapy.

In our benchmark, we included methods based on four different types of features which were *k*-mer counts, percentage-spliced-in (PSI), transcripts per kilobase million (TPM), and sequencing counts. The two latter were measured and tested across annotated genes and transcripts separately. The algorithms we benchmarked were DESeq2 [12], edgeR [13], and limmaVoom [14] for TPM and sequencing counts; iMOKA for *k*-mer counts; and Whippet [15] for alternative splice site usage. We excluded four other *k*-mer based methods HAWK [16], KOVER [17], Kissplice [11], and GECKO [9] because they were respectively impossible to run on such big datasets due to segmentation fault errors, were unable to find *k*-mers that could classify the input samples or, for the last two methods, were killed after 2 weeks of runtime on our computer cluster.

In our benchmark, we compared the list of features output by each algorithm by using them in a random forest classifier and determining their out of bag scores (OOB score). The out of bag score tests how well each classifier performs without having to set aside a portion of the data specifically as a test set. It is as reliable as using a test set [18, 19] without having to set aside part of the data. We chose the random forest classifier because it is a non-parametric approach and because the importance of each input feature is easy to evaluate.

Finally, for the largest dataset, the molecular classification of breast cancer, we performed a 5-fold cross validation of the entire iMOKA procedure and all other benchmarked algorithms, using 4/5 of the dataset for data reduction and creation of a random forest model and 1/5 of the dataset as the test set.

### Classification of breast cancer subtypes

**Fig. 2:**
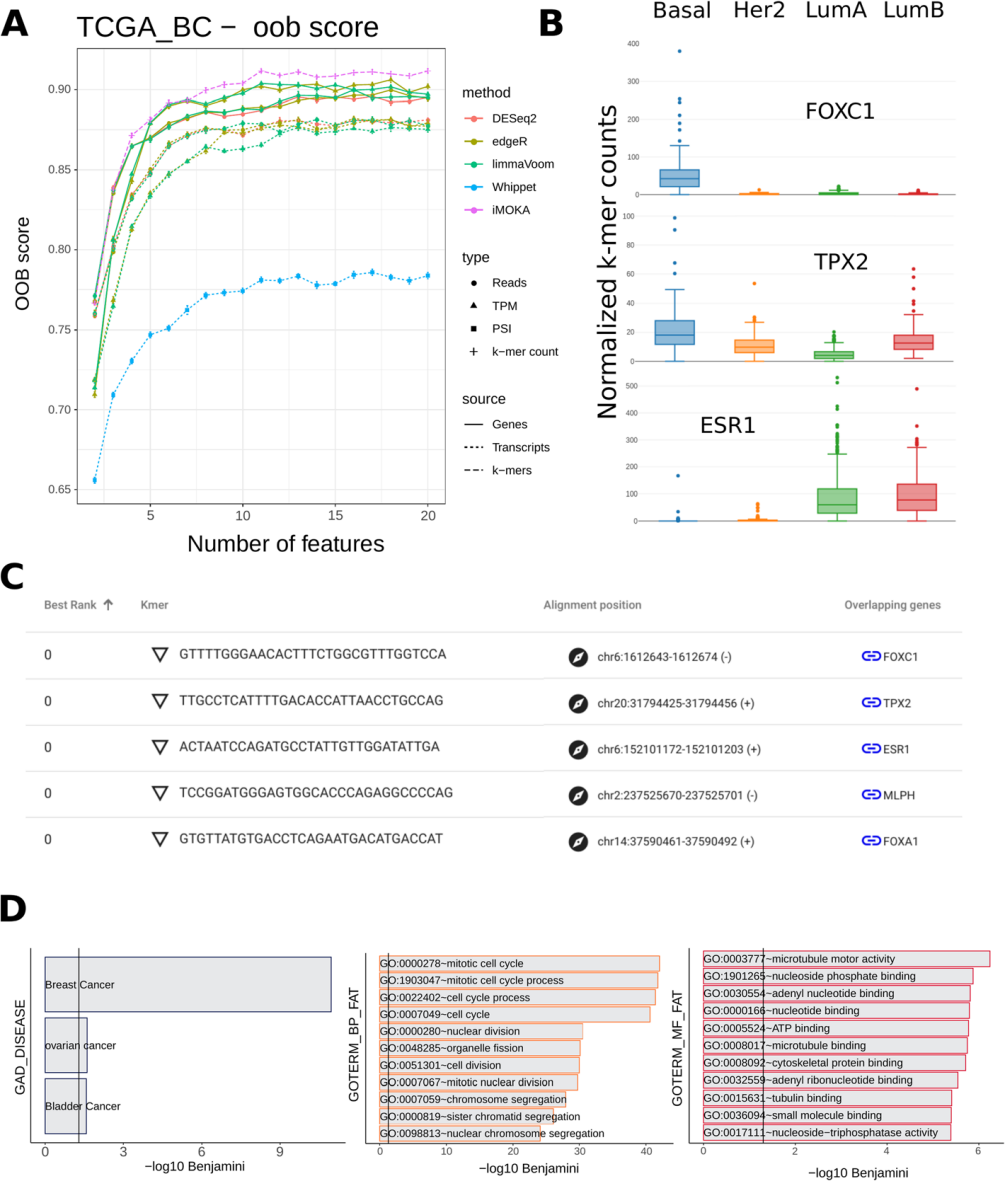
iMOKA accurately predicts breast cancer subtypes. **a** The features output by all benchmarked approaches are evaluated for their capacity to classify breast cancer subtypes using Random forest’s oob score plotted as a function of the number of the best features output by each approach. **b** Screenshot of the iMOKA output with each *k*-mer sequence, their rank in the classification of breast cancer subtypes, and where these sequences map to on the genome. **c** Screenshot of the iMOKA display showing *k*-mer counts of the 3 highest ranking *k*-mers across the 4 subtypes. **d** Gene ontology of the genes overlapping the *k*-mers selected by iMOKA

Breast cancer is a transcriptionally heterogeneous disease with multiple subtypes that determine prognosis, treatment, and patient outcome. Although breast cancer classification is constantly being updated, a broadly accepted stratification defines four groups which are luminal A (LumA), luminal B (LumB), HER2-enriched (HER2), and basal-like [20]. We benchmarked iMOKA on a dataset of 1038 mRNA-Seq breast cancer samples from the Cancer Genome Atlas (TCGA) Pan-Gyn cohort [21] (patients per class: basal 190, Her2 82, LumA 559, LumB 207) and tested how well the outputs of each approach could accurately predict the four classes. We found that the list of *k*-mers output by iMOKA (Additional file 1, Fig. S5) was above all other methods in their ability to classify the four types of breast cancer (Fig. 2a). The worst performing features were the splice site usage statistics given by Whippet. This could be expected because the breast cancer stratifications were originally created using gene expression profiles, not splicing events.

We additionally performed a 5-fold cross validation of the entire iMOKA procedure and all other benchmarked algorithms including feature reduction and model generation. The accuracies of the final models (Fig. S2) show a consistent behavior to the oob scores in Fig. 2a.

iMOKA identified 3002 *k*-mers overlapping different types of events (Table S1 and Additional file 1). Using iMOKA’s interface, we were able to explore the genes to which these *k*-mers mapped (Fig. 2b). As expected, within the best ranking *k*-mers, iMOKA found overlaps with genes that have been extensively linked to breast cancer subtypes and are already used in the clinic such as estrogen receptor 1 (ESR1) [22], Forkhead Box A1 (FOXA1) [23], Forkhead Box C1 (FOXC1) [24], xenopus kinesin-like protein 2 (TPX2) [25], and Melanophilin (MLPH) [26]. By clicking on the *k*-mer sequence in the iMOKA interface, we can visualize the representation of each *k*-mer in the 4 classes (Fig. 2c). The top three *k*-mers, whose gene expression is shown in Fig. S3, have representation profiles that clearly explain iMOKA’s high classification accuracy with a small number of *k*-mers.

It is worth noting that iMOKA picked up 120 potential alternative splicing events. Amongst these were 4 extensively studied splicing isoforms (MYO6, TPD52, IQCG, and ACOX2) [27] identified to be amongst the 5 most important isoforms differentially expressed between ER+HER2− and ER-HER2 primary breast tumors (Fig. S4).

Finally, we used DAVID [28] to perform a functional annotation of the genes overlapping the *k*-mer selected by iMOKA. The gene list is strongly enriched for breast cancer-associated genes and of genes associated with the function commonly dysregulated in cancer cells, such as cell cycle, cell division, and motility (Fig. 2d and Additional file 4).

### iMOKA identifies events associated with the response to treatment in ovarian cancer patients

**Fig. 3:**
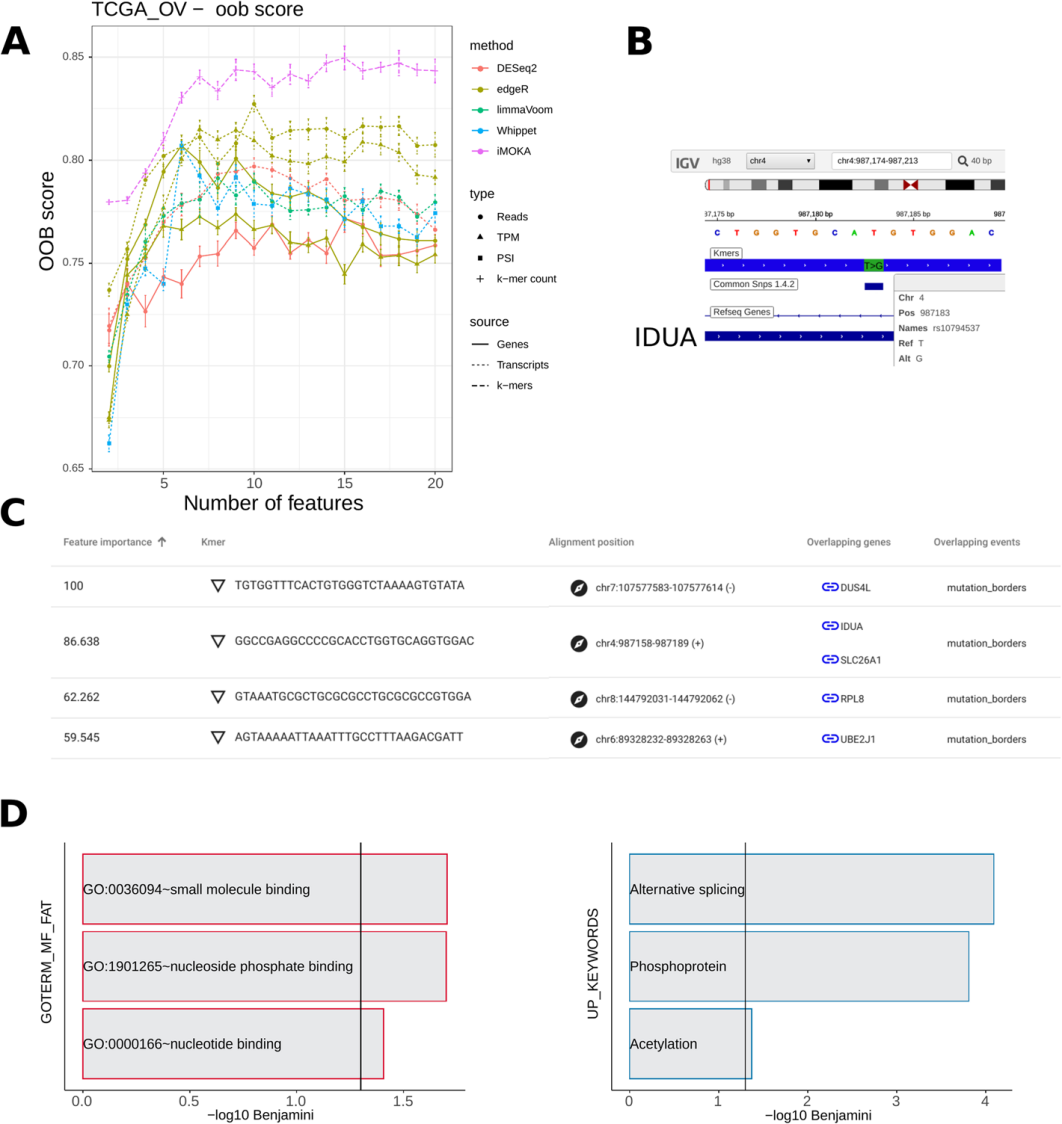
iMOKA accurately predicts response to chemotherapy in ovarian cancer. **a** The features output by all benchmarked approaches are evaluated for their capacity to classify response to chemotherapy. Classification oob score is plotted as a function of the number of features output by each approach. **b** Screenshot of the iMOKA output showing each *k*-mer sequence that maps to a SNV, their importance in the classification, where these sequences map to on the genome, and what genomic feature they correspond to. **c** Screenshot of the IDUA SNV detected by iMOKA. **d** Gene ontology of the genes overlapped by the *k*-mers found by iMOKA

Our second benchmark was performed on a dataset of high-grade serous ovarian cancers taken from the TCGA_OV cohort [29]. We included patients having an annotated [30] response to a first-line treatment to the combination platinum and taxane chemotherapy (patients per class: 174 responsive, 66 non-responsive). iMOKA identified 138 *k*-mers with individual accuracy between 65 and 75% (Table S1 and Additional file 2). Again, the *k*-mers found by iMOKA gave the most accurate oob scores for response to chemotherapy (Fig. 3a). The gain compared to other methods is much higher than for the previous breast cancer classification. This can be explained by the fact that most of the methods we benchmark against only make use of gene or transcript expression or splicing sites. Breast cancer stratification is mainly based on gene expression, and therefore, these methods compare well with iMOKA. However, in the case of response to chemotherapy in ovarian cancer, iMOKA is able to also make use of single nucleotide variants (SNVs) and splice site usage to make its predictions (Fig. 3b). Via the iMOKA interface, we can visualize the SNVs with the highest feature importance. Thus, we can observe that iMOKA detected a known nonsense mutation (SNP id: rs10794537) in the alpha-l-iduronidase (IDUA) gene. IDUA is responsible for the degradation of the mucopolysaccharides, heparan sulfate, and dermatan sulfate which modulate angiogenesis, cell invasion, metastasis, and inflammation [26] and importantly are ligand receptors for polynuclear platinum anticancer agents [27]. In agreement with this, the gene ontology (Fig. 3d) analysis shows a functional enrichment of small molecule binding proteins.

### iMOKA identifies events associated with the response to neoadjuvant chemotherapy in breast cancer patients

**Fig. 4:**
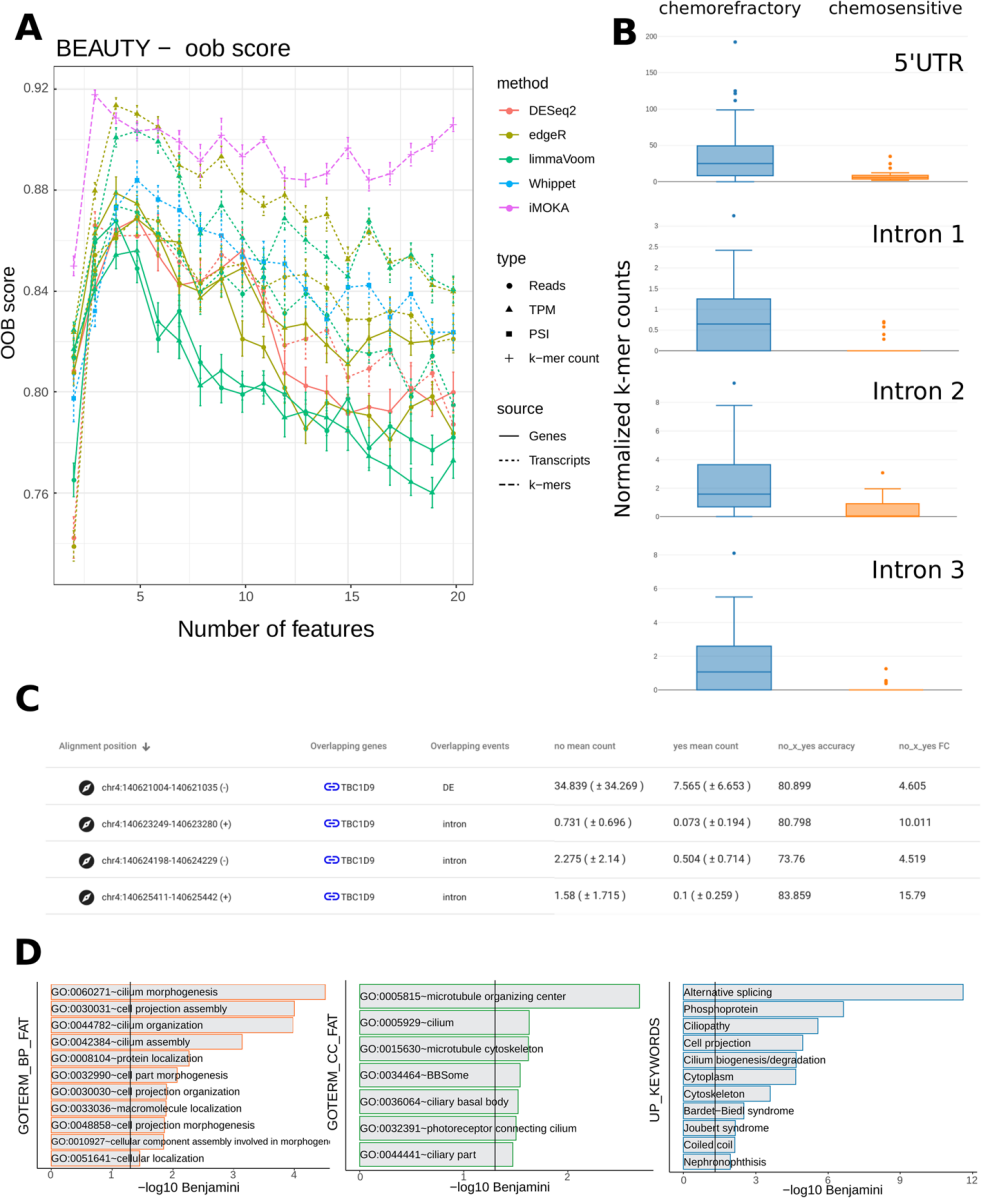
iMOKA identifies novel events that predict response to neoadjuvant chemotherapy in breast cancer. **a** The features output by all benchmarked approaches are evaluated for their capacity to classify response to chemotherapy. OOB scores are plotted as a function of the number of features output by each approach. **b** The *k*-mers abundance of four *k*-mers mapping respectively on the 5′UTR and in the last three introns. **c** Four *k*-mers overlap the gene TBC1D9 in different positions capturing a change in overall gene expression and an intron retention event. **d** Gene ontology of the genes overlapped by the *k*-mers found by iMOKA

The third test dataset was taken from the Breast Cancer Genome Guided Therapy (BEAUTY) study [31] and consisted of patients with all 4 types of breast cancer for which we tested the response to neoadjuvant chemotherapy with paclitaxel and anthracycline. This allowed us to test the binary classification of more heterogeneous cell populations on smaller sample sizes: 36 patients that had a complete response to chemotherapy and 82 that did not. It is worth noting that this dataset presented a significant batch effect, detected using the R package DASC [32], associated with the load date of the samples (Fig. S5). Despite this, iMOKA identified 1248 *k*-mers with an individual accuracy between 70 and 83.8% (Table S1 and Additional file 3). Again, the *k*-mers discovered by iMOKA give the highest oob scores for the response to chemotherapy (Fig. 4a).

Our method can identify multiple events on the same gene that are useful for classification. For example, as shown in Fig. 4b for the highest scored *k*-mers overlapping the gene TBC1D9, iMOKA discovers that the gene as a whole is differentially expressed between conditions but also discovers alternatively expressed introns (Fig. 4c) that were confirmed as being a retained intron using a dedicated algorithm, IRFinder [33].

The gene ontology analysis of the genes overlapping the *k*-mers selected by iMOKA reveals a strong relationship with microtubules and cilia, components influenced by paclitaxel [34, 35], an anti-microtubule agent of the taxane family used as part of the therapy on all the patients in the study. Although the study included heterogeneous cancer types and an unbalanced dataset, iMOKA was able to detect features useful for classification.

### iMOKA identify DE genes associated with DLBCL chemoresistance

**Fig. 5:**
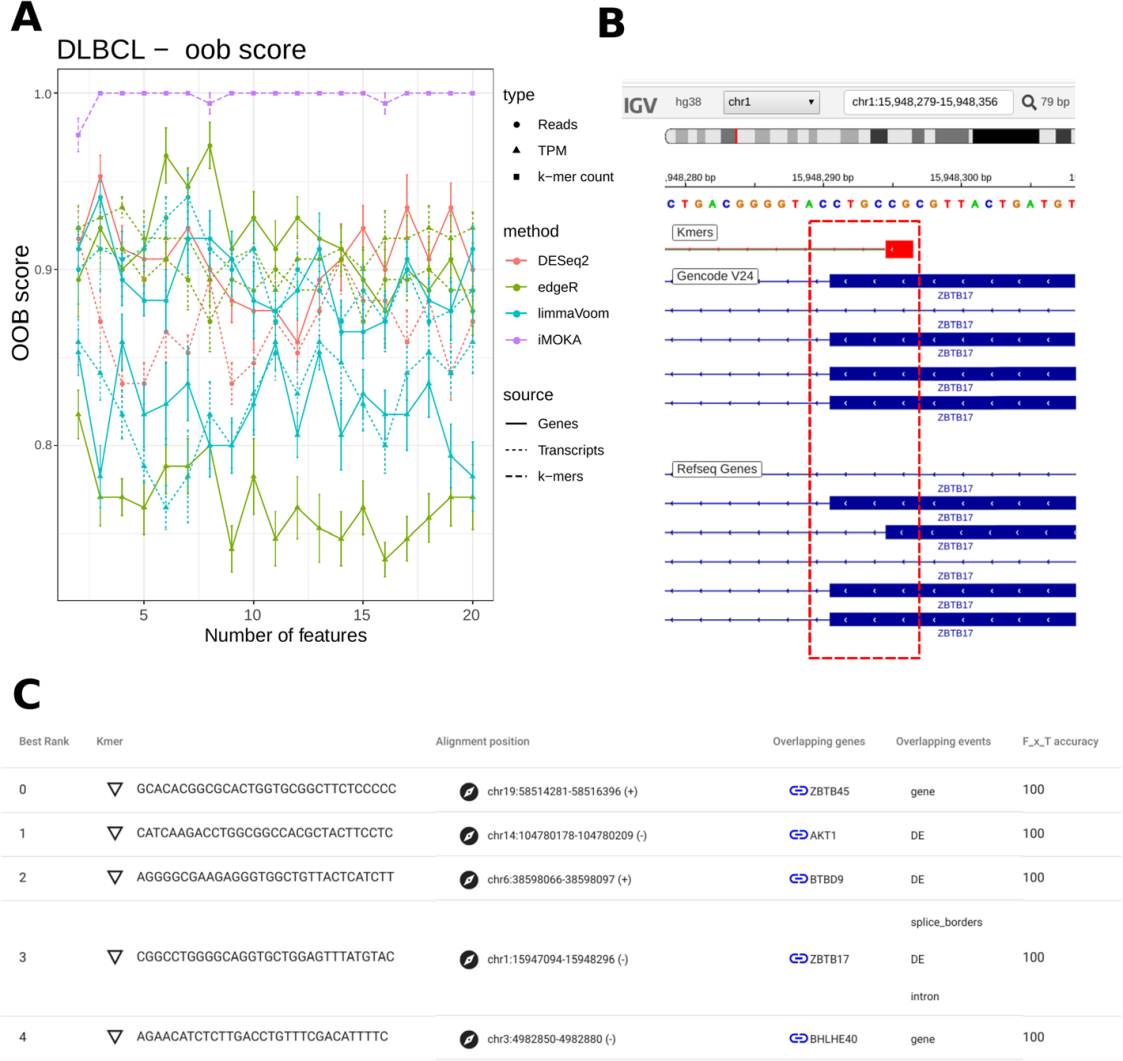
iMOKA identifies DE genes and transcripts between chemorefractory and chemosensitive DLBCL patients. **a** The features output by all benchmarked approaches are evaluated for their capacity to classify response to chemotherapy. RF’s oob scores are plotted as a function of the number of the best features output by each approach. iMOKA reach the highest score thanks to the five *k*-mers with 100% of individual accuracy. **b** iMOKA GUI screenshot showing the detail of the splicing site of the gene ZBTB17, where the isoform NM_001242884 is detected as an event of interest by iMOKA, present in the RefSeq but not in GENCODE. **c** Five *k*-mers able to separate the responsive patients from the chemorefractory ones. *k*-mer normalized counts and the respective gene counts available in Fig. S6

In the last dataset, we tested iMOKA in a frequent scenario where differential representation of transcripts is assessed in a very small cohort. To this end, we considered 17 DLBCL patients [36], 10 responsive to an anthracycline-based regimen R-CHOP (rituximab, cyclophosphamide, doxorubicin, vincristine, and prednisone) and 7 non-responsive. The RNA-seq used for this dataset is targeted, making it impossible to evaluate the PSI values, so only the abundance of the genes and transcripts were considered in the benchmark (Fig. 5 and Fig. S7). iMOKA identified 1928 *k*-mers having an individual accuracy over 80% and five with 100% accuracy. They corresponded to the genes AKT1, BTBD9, ZBTB45, ZBTB17, and BHLHE40. Amongst those, AKT1 is known to play a role in DLBCL chemosensitivity [37] but was not detected as differentially expressed in the original publication [36].

This study highlights another advantage of using *k*-mers; they are agnostic to transcript annotation. For example, the *k*-mer overlapping ZBTB17, a gene involved in B cell development and differentiation [38], is located on the splicing site at position chr1:15,947,123-15,948,295 and is part of Refseq transcript NM_001242884. However, this transcript was not annotated in the GENCODE annotation (Fig. 5b) and thus not detected by salmon.

### iMOKA runtimes and disk space

**Fig. 6:**
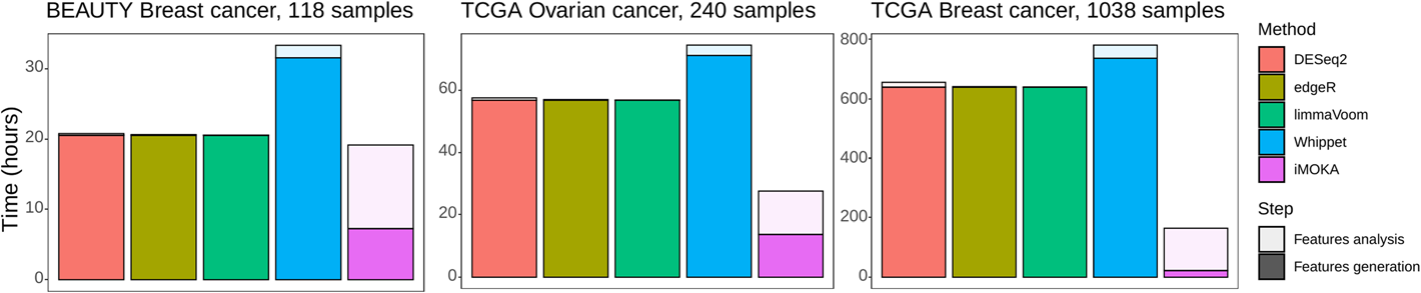
iMOKA is faster and scales better with large cohorts. Comparison of the running times between the benchmarked methods. Solid bars represent the time dedicated to the generation of the features (transcript abundance, PSI evaluation, and *k*-mer count), and the lighter bars represent the time dedicated to the analysis of the features (differential expression, differential splicing, and the machine learning-based filters)

iMOKA was designed to be scalable; the user can control the number of threads used and the dedicated RAM, allowing the software to run not only on HPC clusters, but also on a laptop. In Fig. 6, we report the times to analyze three experiments described in the previous sections on a computer with 8-cores and 32 GiB of RAM. Importantly, the higher the number of samples in the cohort, the bigger iMOKA’s gains are.

iMOKA’s most intensive task is the generation of informative *k*-mers, where a large amount of data is filtered and aggregated, while the other benchmarked approaches handle data that are already filtered (reads are already mapped to annotated regions). Finally, most methods that calculate differential expression are designed for relatively small cohorts and do not scale well in memory with large cohorts: DESeq2 and edgeR for example required additional RAM in order to analyze the differential expressed transcripts in the TCGA BRCA (TCGA_BC) analysis (61 GiB and 46 GiB, respectively) (Fig. 6).

## Discussion

Recent efforts to aggregate and annotate patient HTS data should facilitate our understanding of health trajectories through multiple molecular mechanisms. In theory, combining gene expression, isoform usage and single nucleotide variation should allow for more nuanced stratification and prediction of disease etiology. However, HTS data analysis often requires extensive data transformations that are often performed with little transverse coherence; each type of analysis produces lists of features that pass a given test and these are then analyzed separately. Mapping to a reference, using ad hoc statistical thresholds for each type of analysis, and grouping sequences by functional elements are common steps in bioinformatics pipelines that may not reflect the complex interaction between each of the processes that make up an individual’s transcriptome.

We designed iMOKA with the aim of analyzing HTS data in the reverse manner; we wished to first discover all sequences that were informative, group them according to how well they could classify the input samples, and then break them down into the different components of gene expression, isoform representation, and SNV presence. In doing so, we created a classifier that could explore HTS data without a reference genome or transcriptome and without the need of dedicated bioinformatics pipelines for each type of transcriptional event.

Using *k*-mer counts removes the requirement of a mapping step and allows iMOKA to explore and combine multiple transcriptional events to make more accurate predictions and to explore all these events simultaneously without having to apply multiple pipelines. *k*-mers can measure changes in transcription, isoform abundance, and sequence simultaneously and were thus able to create better predictive models than other metrics such as transcripts per million (TPM), read counts, or splice site usage.

By creating a reliable, cross-platform user interface, iMOKA allows non-specialists to leverage the predictive power of our approach in a manner that is fast and accurate. In addition, iMOKA uses a flexible data structure that allows the easy integration of new samples and uses only a fraction of the disk space required for stocking compressed sequencing files. In addition, *k-mer* based approaches such as iMOKA have the advantage of being portable; *k-mer* sequences will not change with new versions of the genome. This is crucial for the integration of omics data with other clinical data such as imaging or patient file records.

## Methods

### Preprocessing

The input data can be given as SRR identifier, BAM, FASTA, or FASTQ files. In the first and second cases, the corresponding FASTQ files are automatically generated using sra-tools’ fastq-dump [39] and SAMtools [40], respectively. If the data is stranded paired end sequencing, the user can reverse complement one or both the files using SeqKit [41]. In order to assert the quality of the FASTQ files, the user can use FASTQC [42] by adding the flag “-q”.

For each sample, KMC3 [9] is used to count the *k*-mers of the length chosen by the user (default *k* = 31). Its output is converted into a sorted binary file optimized for the following steps of iMOKA and a JSON file containing the metadata information.

The binary file is divided into two parts: a suffix portion, containing the nucleotidic sequence and the relative count, and a prefix portion, which contains the prefixes and the positions of the respective suffixes.

The length of the prefix is defined using the following formula, an adaptation from [43]:

$$ p=0.5\times {\log}_2(t)-0.5\times {\log}_2\left({\log}_2(t)\right) $$

where *p* is the prefix size and *t* is the total number of different *k*-mers for the current sample.

### Matrix generation

The input to the feature reduction step is a JSON file containing the name, group, and localization of the sorted binary *k*-mer count file of each sample in the analysis. The JSON file also stores the sum of all the *k*-mer counts that will be used as a normalization factor:

$$ {N}_{ij}={C}_{ij}\times \frac{\mathrm{RF}}{T_j} $$

where

*N*_*ij*_ is the normalized count of the *i*th *k*-mer of the sample *j*

*C*_*ij*_ is the raw count of the *i*th *k*-mer of the sample *j*

*T*_*j*_ is the sum of the counts of all the *k*-mers of the sample *j*

RF is a rescaling factor, used to increase the value of all the normalized values and avoid computational problems related to precision. By default, RF = 1e9

Each thread starts the creation of the matrix and the reduction step in parallel, using an OpenMP [44] implementation, at a different point of the matrix according to the number of threads available using the following formula:

$$ {K}_t=\frac{4^k-1}{T}\times t $$

where

*T* is the total number of threads available

*K*_*t*_ is the first *k*-mer analyzed by the thread *t* (from 0 to *T* excluded) considering all the possible ordered combination from 0 to 4^*k*^

*k* is the length of the *k*-mers (default 31)

The last *k*-mer analyzed by each thread is *K*_*t* + 1_ – 1. For example, with 2 threads (*T* = 2) and *k* = 31, the first *k*-mers for each threads will be:

$$ {K}_0=\frac{4^{31}-1}{2}\times 0=0= AAAAAAAAAAAAAAAAAAAAAAAAAAAAAAA $$

$$ {K}_1=\frac{4^{31}-1}{2}\times 1=2305843009213693952= GAAAAAAAAAAAAAAAAAAAAAAAAAAAAAA $$

Finally, the buffer size reserved for each sample is dependent on the number of parallel processes, the number of total samples, and the available memory reserved:

$$ buff=\frac{{\mathrm{RAM}}_{\mathrm{avail}}}{\alpha \times N\times T} $$

where

*Buff* is the length of the buffer

RAM_avail_ is the available RAM in GiB, defined by the user using the environmental variable “IMOKA_MAX_MEM_GB”

*N* is the number of samples in the matrix

*T* is the total number of threads available

*α* is a factor representing the GiB occupied by 1000 *k*-mers, approximated to 0.011

### Bayesian classifier *k*-mer accuracy assessment

The accuracy of each *k*-mer is calculated using the NaiveBayesClassifier method implemented in the library mlpack [45]. For each *k*-mer, the samples are randomly divided into test and training sets, with an equal number of samples for each group scaled to the smallest one:

$$ {n}_{\mathrm{test}}=\mathrm{round}\left({n}_{\mathrm{min}}\ast {p}_{\mathrm{test}}\right) $$

$$ {n}_{\mathrm{train}}={n}_{\mathrm{min}}-{n}_{\mathrm{test}} $$

where:

*n*_min_ is the dimension of the smallest group

*n*_test_ and *n*_train_ are respectively the dimension of the test and training sets

*p*_test_ is the test fraction, 0.25 by default

Using one feature (*k*-mer count) *x*_*k*_ at a time, the NaiveBayesClassifier class computes for each label *y*_*j*_:

$$ P\left(X={x}_k\vee Y={y}_j\right) $$

$$ P\left(Y={y}_i\right) $$

Given that we use a pairwise comparison with a constant number of training samples amongst the labels, all the *N*_labels_ have the same probability

$$ P\left(Y={y}_i\right)=P\left(Y={y}_{j+1}\right)=\frac{1}{N_{\mathrm{labels}}} $$

The label prediction of a sample *i* based on the *k*-mer count *x*_*k*_ is then given by:

$$ {y}_i= argmax\left(P\left(Y=y\right)\right) $$

The accuracy of the *k*-mer *k* is computed considering only the samples part of the test set:

$$ {acc}_k=\frac{T}{n_{\mathrm{test}}}\times 100 $$

where

*acc*_*k*_ is the accuracy of the *k*-mer *k*

*T* is the number of correct labels assigned in the test set

Because the accuracies depend on the random division of the training and test sets, we use a Monte Carlo cross validation [46] with a given number of iterations ( -c argument, default 100). This cross validation can be ended by a conditional break that is triggered when the standard error across iterations drops beneath a given threshold ( -s argument, default 0.5).

The *k*-mers that achieve an accuracy higher than the accuracy threshold (-a argument, default 65) in at least one of the pairwise comparisons are saved in a text file, along with the accuracy values.

### Entropy filter booster

In order to speed up the process of accuracy estimation, we introduced an additional filter based on the Shannon entropy [47] of the counts of each *k*-mer that runs in parallel to the Bayesian filter (BF).

For a given *k*-mer *k* and its counts in the different samples *C*_*k*_ = (c_*k*0_, c_*k*1_, ... c_*kn*_), we compute its entropy value *H*_*k*_ as follows:

$$ {H}_k=-{\sum}_{i=0}^n{f}_{ki}\times {\log}_2\left({f}_{ki}\right) $$

$$ {f}_{ki}=\frac{c_{ki}}{\sum_{j=0}^n{c}_{kj}} $$

The filter uses an adaptive threshold, *H*_thr_, tuned according to the lowest entropy detected in the previous batch of *k*-mers that passed the accuracy filter (*H*_min_).

Initially *H*_thr_ = 0, so all the *k*-mers in the first batch are evaluated by the BF and the lowest entropy is saved as *H*_min_. During the analysis, *H*_thr_ is updated when more than *E*_up_ (initially equal to 30) passes the BF. The first assignment is always:

$$ {H}_{\mathrm{thr}}={H}_{\mathrm{min}}-\left({H}_{\mathrm{min}}\times {a}_1\times 2\right) $$

Subsequently:

$$ \mathrm{IF}\kern0.28em \left({H}_{\mathrm{thr}}>{H}_{\mathrm{min}}-\left({H}_{\mathrm{min}}\times {a}_1\right)\right): $$

$$ {H}_{\mathrm{thr}}={H}_{\mathrm{min}}-\left({H}_{\mathrm{min}}\times {a}_1\right) $$

$$ \mathrm{ELSE}: $$

$$ {H}_{\mathrm{thr}}={H}_{\mathrm{min}}+\left({H}_{\mathrm{min}}\times {a}_2\right) $$

The adjustment parameters *a*_*1*_*» a*_*2*_ ensure that the new threshold is not set too close to the minimum *H*_min_.

The number of *k*-mers required to update the threshold (*E*_up_) increases by 30 at each update in order to reduce the number of computations and reduce the fluctuations of the threshold. Figure S1 shows the entropy in function of the BF estimated accuracy of a sample of *k*-mers from the previously defined datasets showing that the number of *k*-mer would have been rejected by the entropy filter but would have had an accuracy higher than 60% are rare and that the adaptive threshold is able to find a mild cutoff that can save more than 50% of the computation, like in TCGA BC, or can let the BF evaluate most of the *k*-mers in case of difficult datasets, like in BEAUTY.

### *k*-mer graph generation

The *k*-mers that successfully passed the reduction are used as nodes in a graph. A link between two nodes is created if they overlap by a minimum number of nucleotides defined by parameter *w* (default = 1). This parameter can be increased if the user notices multiple small sequences in the final result, caused usually by *k*-mers with accuracy close to the given threshold arguments -T and -t, respectively the minimum accuracy required to consider a *k*-mer in the graph construction and the minimum accuracy required to generate a sequence from a graph.

iMOKA then prunes short bifurcations in the graph where there is only one node following the bifurcation. If there are multiple sequential bifurcations, then the branch with the lowest accuracy is removed.

The accuracy values are then rescaled from 0 to 100 for each pairwise comparison in order to normalize the accuracy values and favor the features that are able to classify pairs of classes that are more difficult to separate.

Since each bifurcation could correspond to a biological event such as a point mutation or splicing isoform, each separate path that results from a bifurcation will be kept as a separate sequence for downstream analysis using a depth-first graph traversal approach. When the traversal meets a bifurcation, the branch having the most similar accuracies values to the bifurcating node is kept in the current sequence and others will generate new sequences. Furthermore, to maintain the context of the bifurcations, three *k*-mers preceding the bifurcation are added to each of those new sequences.

### Graph mapping and annotation

The sequences generated from the graphs can be aligned to a reference genome. Currently, iMOKA supports any aligner that provides an output in SAM or pslx format and uses the information given in the JSON configuration file “mapper-config” (-m argument) to align and to retrieve the annotation file, in GTF format. In this manuscript, we used gmap v. 2019-05-12 with the human genome GRCh38 and the GENCODE annotation v29, excluding from the file the entries with the transcript type “retained_intron”.

Once the *k*-mer graphs are aligned, iMOKA identifies the following “alignment derived features” (ADF):

- Mutations, insertions, deletions, and clipping are identified by the letters “M”, “I”, “D” and “S,” respectively, in the alignment’s CIGAR string.
- Alternative splice sites are identified when a *k*-mer graph is split across exons.
- Differential expression (DE) is identified if 50% (set by parameter d) of an annotated transcript is covered by the *k*-mer graphs. Since regions with sequence variations not associated with the classes generate holes in the graphs reducing the portion of the transcripts that generate useful *k*-mers, a higher threshold might result in classifying DE event as general “gene” event, that is, the best *k*-mer in a gene.
- Alternative intronic events are identified if 50% (set by parameter d) of an annotated intron is covered by the *k*-mer graphs.
- Intergenic events are identified if the *k*-mer graph maps to the genome but not to any annotated transcript.
- Unmapped or multimapped events are created for those *k*-mer graphs that have no mapping or map to multiple sites.

iMOKA will preserve one *k*-mer per event, the one with the highest accuracy score. Table S2 contains the list of events with a detailed description.

### iMOKA implementation

The feature reduction component of iMOKA is implemented in C++ using the following libraries: MLpack [45], armadillo [48], cephes [49], cxxopts [50], and nlohmann/json [51]. The self-organizing map and the random forest are implemented in python 3 using the following libraries: numpy [52], pandas [53], sklearn [54], and SimpSOM [55]. The whole software is included in a ready-to-use Docker and Singularity [56] image and is released under the Open Source CeCILL license.

### Benchmark

Transcript abundance was computed using Salmon [57] version 1.1.0 using the index built on the reference transcriptome GENCODE v29 (hg38). The PSI values were computed using Whippet [15] version v0.10.4. We processed the samples in parallel in 4 processes allowing 2 threads and a maximum of 8 GiB of RAM each. The differential expression analysis was performed between each pair of classes in R v3.6.3 using the parameters and functions described in a recent benchmark [58] for the methods DESeq2 [12], edgeR [13], and limmaVoom [14]. Significantly different PSI values between two subsets were detected using whippet-delta.jl, included in the Whippet package.

### Random Forest classifier feature selection and oob score comparison

In order to compare the same number of features extracted by each pipeline, we used the sklearn method SelectFromModel to select 20 features using a decision tree classifier (DTC) trained with all the samples and all the features in order to identify twenty features that, in combination, can be good classifiers. Using an increasing number of features, from 2 to 20, we trained multiple RandomForestClassifier to retrieve the out of the box scores.

We also performed a 5-fold cross validation of the largest and better characterized dataset, TCGA BRCA, to evaluate the accuracy of a model on unseen data. For each fold, we performed the feature reduction using only the training in each method. The final list of features is reduced similarly as for the oob score determination and the balanced accuracy score is estimated for the test set.

## Supplementary information

**Additional file 1.** TCGA_BC_aggregated.json - iMOKA results for the dataset TCGA_BC.

**Additional file 2.** TCGA_OV_aggregated.json - iMOKA results for the dataset TCGA_OV.

**Additional file 3.** BEAUTY_aggregated.json - iMOKA results for the dataset BEAUTY.

**Additional file 4.** DLBCL_aggregated.json - iMOKA results for the dataset DLBCL.

**Additional file 5.** GO - folder containing the DAVID gene ontology result for each dataset.

**Additional file 6.** iMOKA_supplementary.docx - Supplementary materials.

**Additional file 7.** Supplementary Figures S1-S7.

**Additional file 8.** Review history.

## Data Availability

The data used in this manuscript are available from the Cancer Genome Atlas under the project ID TCGA-BRCA [21] and TCGA-OV [29] with dbGaP study accession identifier phs000178.v11.p8 [59]; the BEAUTY dataset [31] is available under the dbGaP study accession identifier phs001050.v1.p1 [59]. Restrictions apply to the availability of these data, which were used under license for those studies, and so are not publicly available. Data are however available by submitting a request to the respective repositories. The DLBCL targeted RNA-seq data [36] are publicly available in the EMBL-EBI ArrayExpress with the accession number E-MTAB-6597 [60]. iMOKA is available at https://github.com/RitchieLabIGH/iMOKA [61] under the Open Source CeCILL license. The copy of the scripts used for the benchmark is available under the subfolder https://github.com/RitchieLabIGH/iMOKA/paper_codes. The DOI for the source version used in this article is 10.5281/zenodo.4008947 [62].
