# Supplementary material for: iMOKA: k-mer based software to analyze large collections of sequencing data
Source: Genome Biol. 2020 Oct 13;21:261. doi: 10.1186/s13059-020-02165-2 (PMC7552494; doi:10.1186/s13059-020-02165-2)
Supplement: Supplementary file 6 — Additional file 6. iMOKA_supplementary.docx - Supplementary materials. [file 13059_2020_2165_MOESM6_ESM.docx]

# **iMOKA is a 𝑘-mer based software to analyze large collections of sequencing data: Supplementary Material.**

### **The data files for each of the 4 benchmark experiments described in the paper are available as Supplementary data in .json format and can be explored with the iMOKA software.**

### **Supplementary Methods**

### **𝑘-mer list and genome browser visualization**

A graphical user interface (GUI) allows the user to visualize the information for each 𝑘-mer, and where the 𝑘-mer graphs map using the javascript implementation of IGV[^1^](https://www.zotero.org/google-docs/?E6tzKb). All the graphs present in the software are generated using the javascript implementation of Plotly[^2^](https://www.zotero.org/google-docs/?i86WFf).

The GUI is implemented in Electron[^3^](https://www.zotero.org/google-docs/?iqS4fe) and in Angular[^4^](https://www.zotero.org/google-docs/?gR6uFe), making it available on multiple platforms (Linux, Windows and MacOS). iMOKA can be run from the interface, in local or on a SLURM[^5^](https://www.zotero.org/google-docs/?StWnPa) cluster, with the only dependency of Singularity[^6^](https://www.zotero.org/google-docs/?jR4k7Y).

**𝑘-mer clustering for samples visualization and clustering**

To visualize the differences and similarities in 𝑘-mer expression between the samples, iMOKA uses a self-organizing map (SOM) to cluster the filtered features.

In the SOM space the features are grouped in nodes by similarity of expression across the samples and similar node are closer in the map space, thanks to the somatotopic capacities of SOM network. We then project a sum of expression for each sample, that is used to visualize the behaviour of the given categories, extracts outliers, or subgroups.

In practice, networks of different size (-n argument) are trained for 1000 iterations (-i argument) to group 𝑘-mers with similar count across the samples. The projections of the 𝑘-mer counts for each sample is used as new set of reduced features, whose importances are evaluated using an extra tree classifier with 2500 trees and their ability to classify is evaluated by cross validation using a linear support vector machine model (-ct argument, the user can choose among 8 different Machine Learning models). Finally, the software uses the aggregated features in a SOM to perform an unsupervised clusterization of the samples (-cs argument to indicate one or more cluster size). The colour coded representation of the SOM projections of each sample and of the averages for each given group can be displayed in the iMOKA interface, thanks to the JSON output files, and also in a standalone HTML pages, making this module an independent tool that can be used on any type of feature.

### **Random Forest Classifier model generator**

iMOKA uses a random forest classifier (RFC) to assign importance to each feature. It also uses a RFC to produce prediction models. Feature importance is estimated with a random forest with 1000 estimators (-n argument) and with min_samples_split (minimum number of samples required to split a node in a tree) of 0.05 (5% of the total number of samples). In order to identify a subset of synergic features, a decision tree classifier (DTC) is trained with all the samples and all the features. The 10 features (-m argument) with the highest feature importance in the DTC are used to produce the final RFC model. If the DTC has less than 10 features, other DTC are created with different seeds. The RFC parameters are chosen using a cross validated grid search on the following values:

- n_estimators: 10, 100 or 500
- min_samples_split: 0.05, 0.10 or 0.15

These parameters can be modified in the random_forest.py script.

All the metrics are evaluated with Monte Carlo cross validation in a similar procedure as described in the reduction step.

**Supplementary Figures**

**
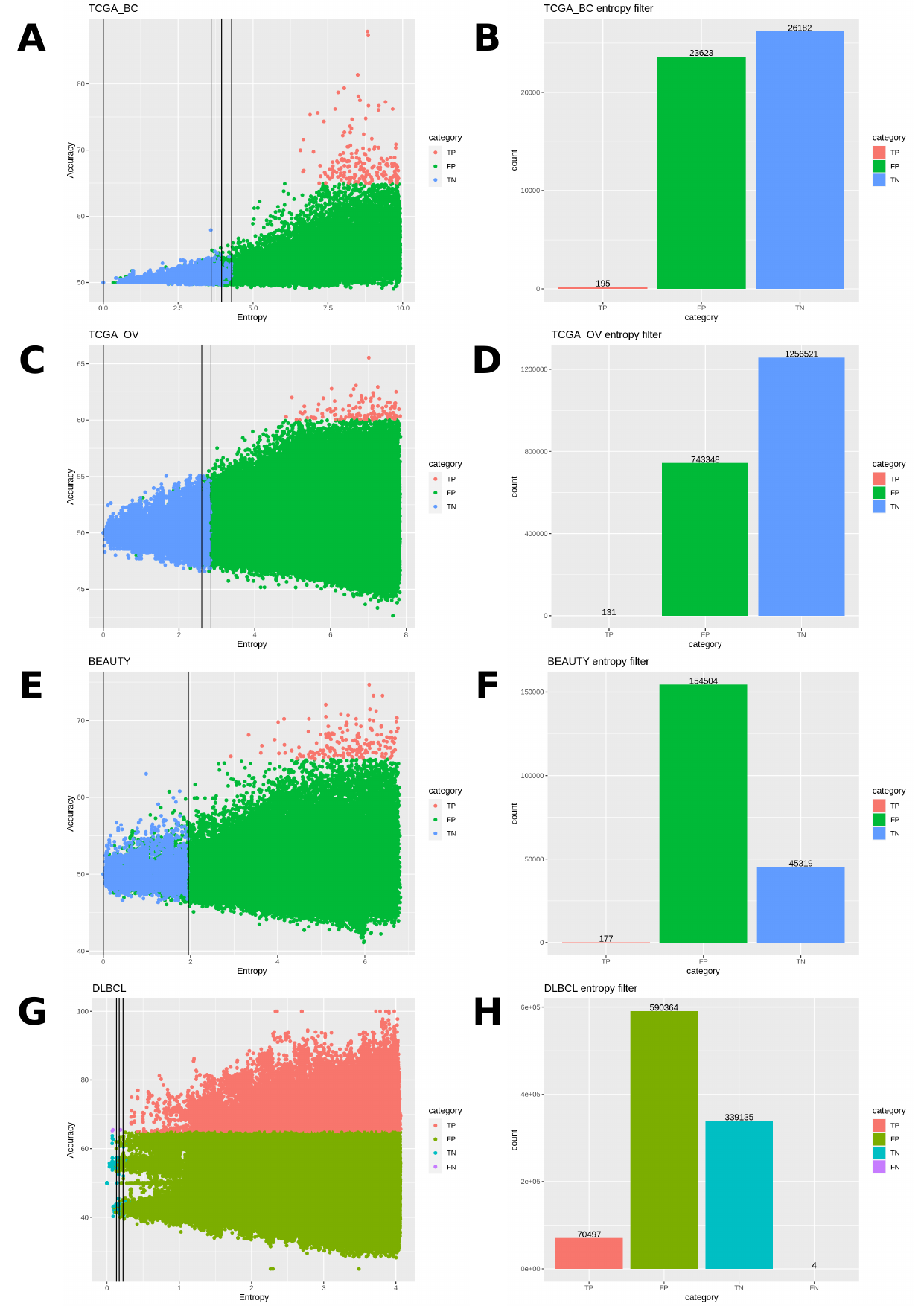
**

**Supplementary Figure 1:** 𝑘-mer count entropy in function of the accuracy estimated by the Bayesian classifier. Both the entropy and the accuracy were computed for samples of 50000, 1000000, 200000 and 1000000 𝑘-mers respectively in TCGA BC (**A-B**), TCGA OV (**C-D**), BEAUTY (**E-F**) and DLBCL (**G-H**) datasets. This graph feature is available in iMOKA_core reduce step using the “-v” argument. **TP**: 𝑘-mers that passed both the entropy filter and the accuracy filter. **TN:** 𝑘-mers discarded by both filters. **FP:** 𝑘-mers that passed the entropy filter but are discarded by the accuracy filter. **FN:** 𝑘-mers discarded by the entropy filter but passed the accuracy filter. The vertical black bars correspond to the values of the adaptive threshold. In DLBCL the first, second and third quartile of the thresholds are represented for clarity. The accuracy thresholds were set to 65 for every dataset except TCGA_OV where it’s of 60 due to the scarcity of positive 𝑘-mers.


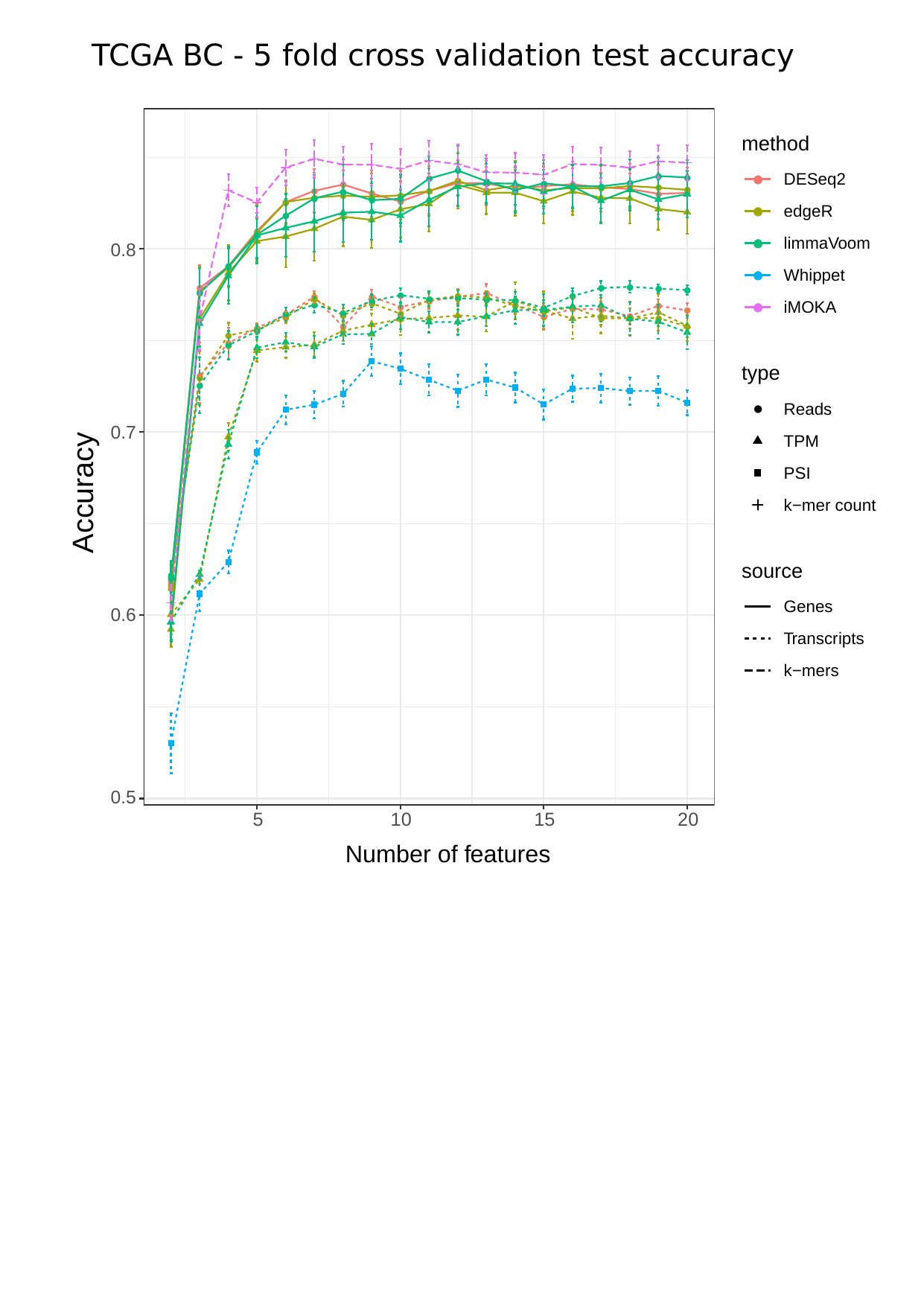


**Supplementary Figure 2:** 5 fold cross validation of the entire iMOKA pipeline on the TCGA BRCA dataset.For each fold, a test set is put aside at the start, before the feature reduction step.


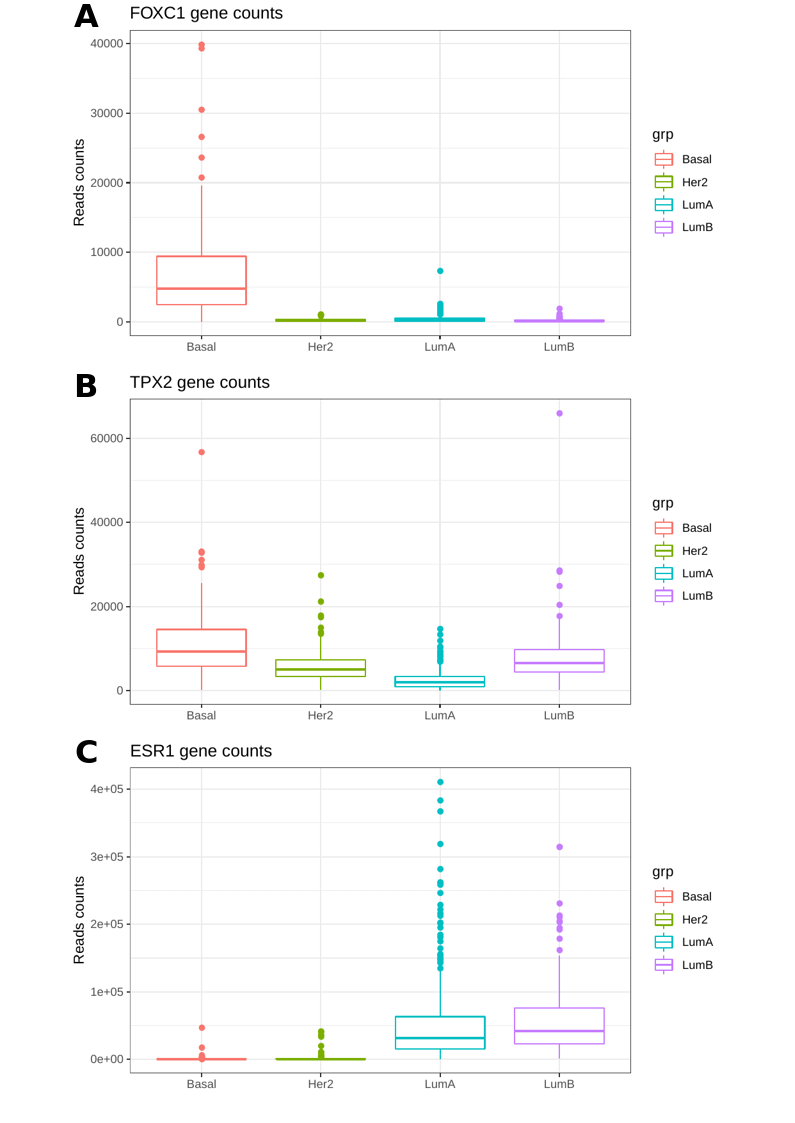


**Supplementary Figure 3:** Read counts of the genes FOXC1 (**A**), TPX2 (**B**) and ESR1 (**C**) in TCGA BRCA, whose corresponding overlapping 𝑘-mers abundances are visible in Figure 2.

**
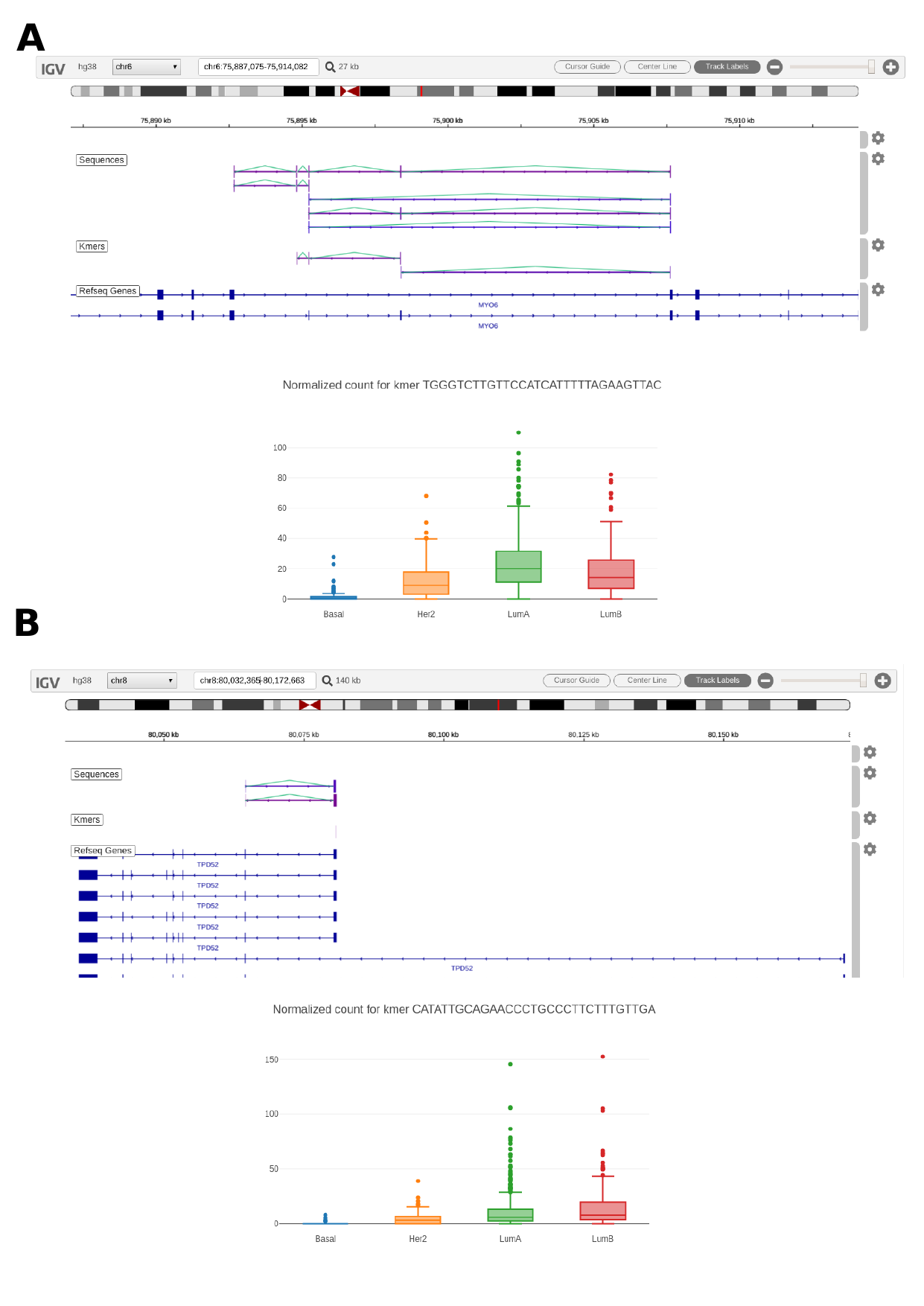

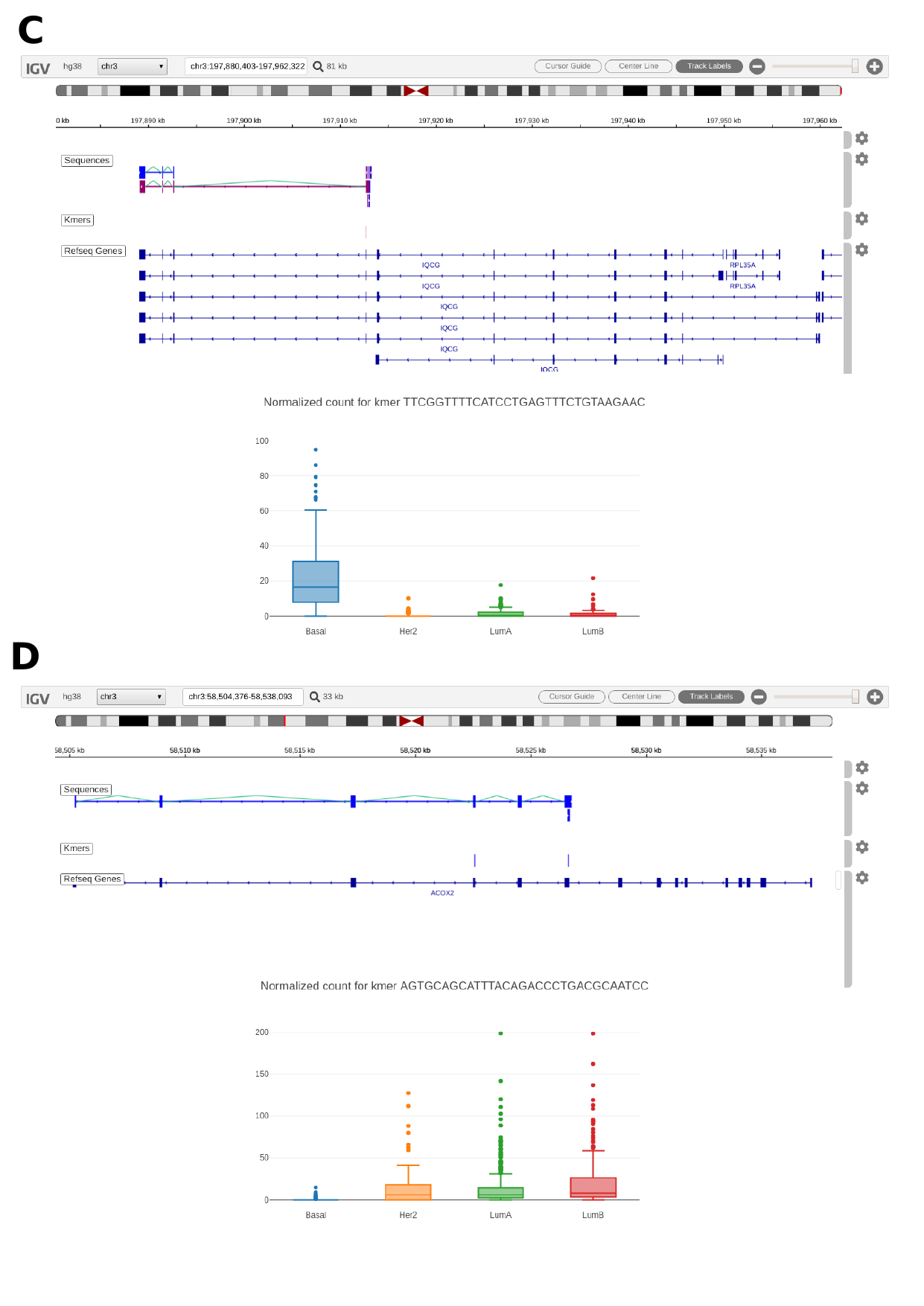
Supplementary Figure 4:** IGV genome browser visualization (integrated in the iMOKA interface) of the 𝑘-mers that map to splicing sites and exons known to be involved in different breast cancer molecular subtypes and the abundances of the representative 𝑘-mers, validated in a recent study[^7^](https://www.zotero.org/google-docs/?z3z0T6). **A**) The inclusion of the exon chr6:75898373-75898410 (hg38) in the gene MYO6. **B**) The first exon in position chr8:80080315-80080830, that is included in 5 out of 12 possible transcripts of the gene TPD52 (GENCODE V.24). **C**) The last four exons forming a transcript with an intronic start site in the gene IQCG. **D**) The last six exons forming a transcript with an intronic start site in the gene ACOX2.
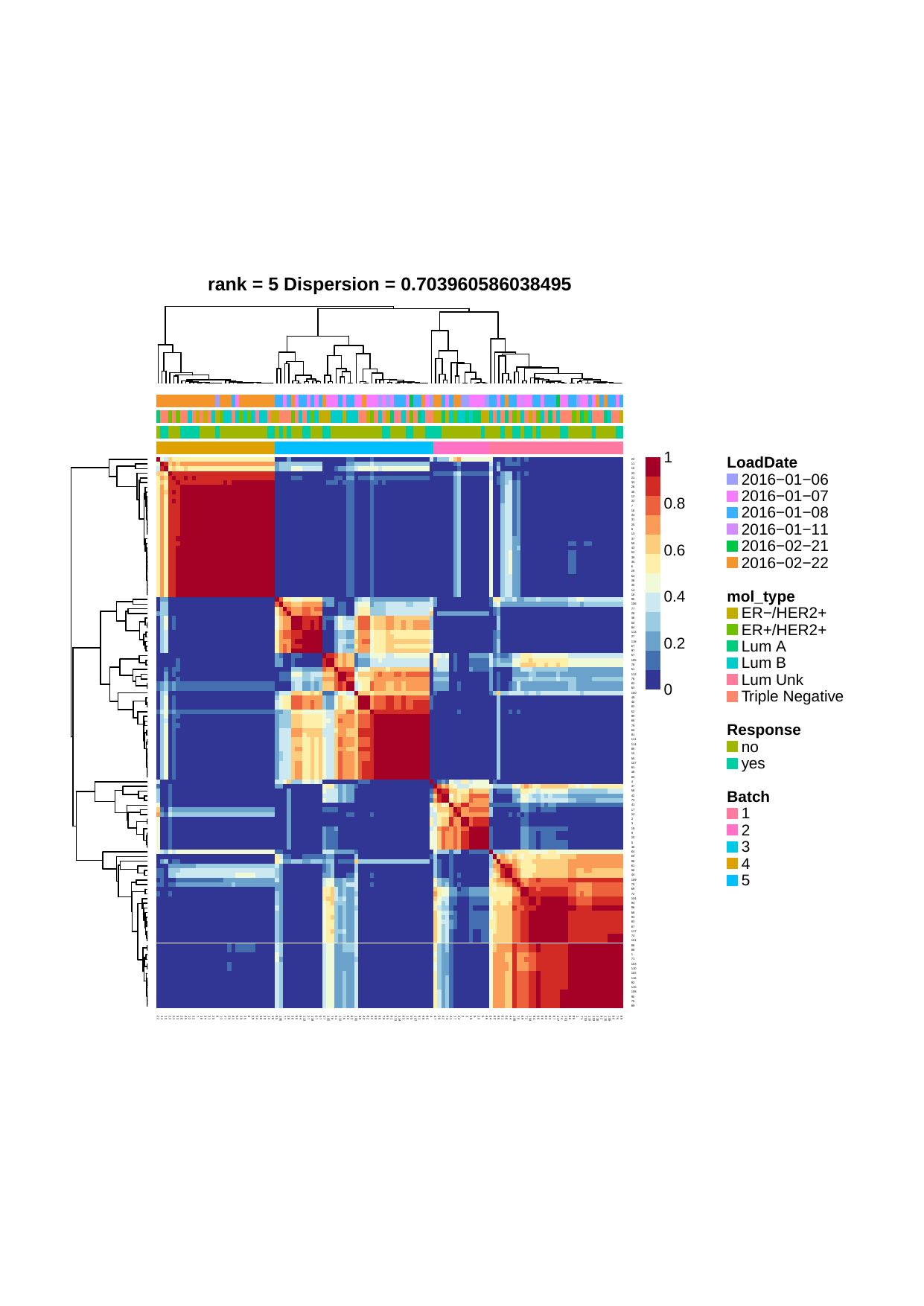

**Supplementary Figure 5:** identification of a hidden batch effect in BEAUTY dataset associated with the Load Date using DASC [^8^](https://www.zotero.org/google-docs/?NTngAb).


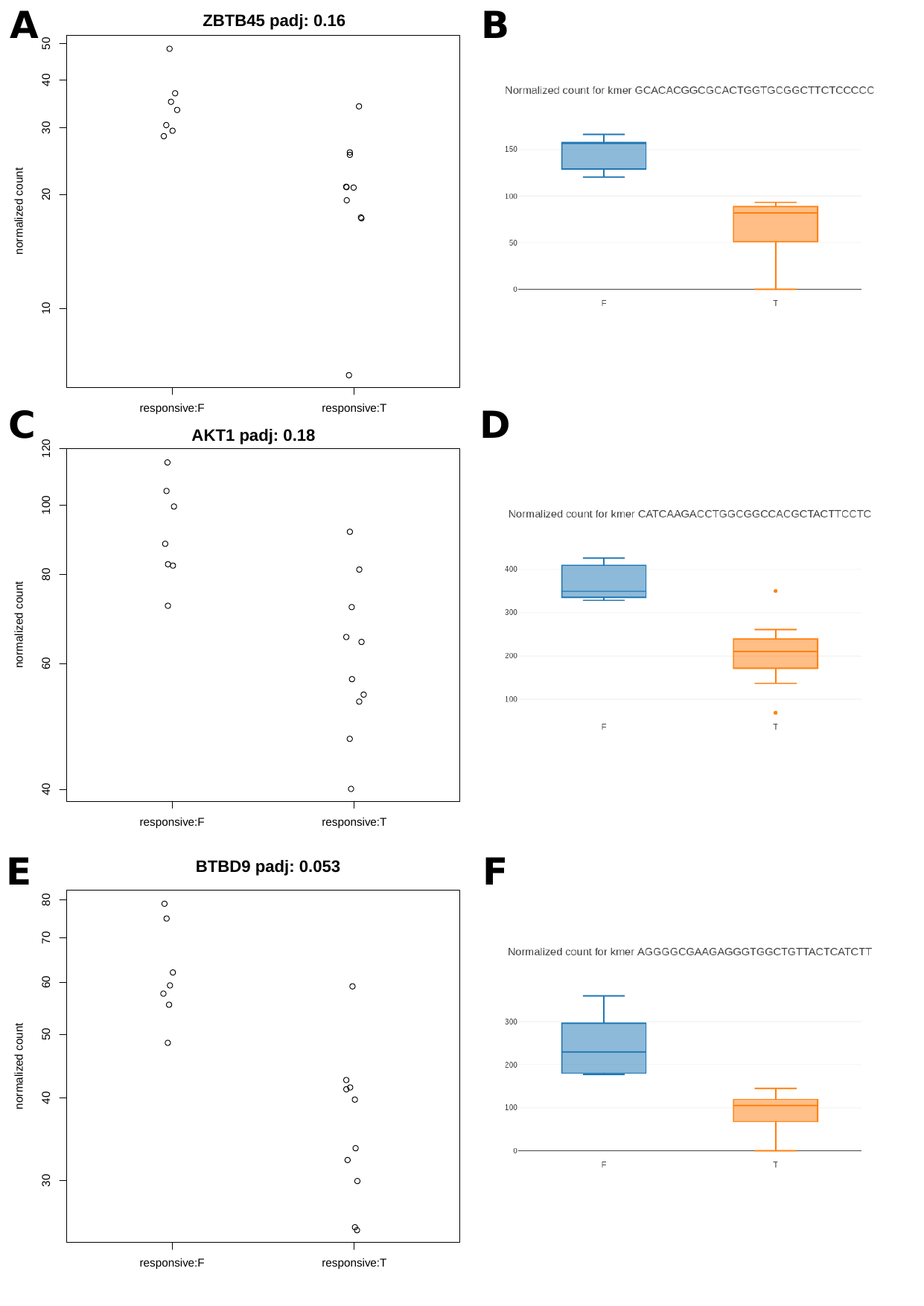


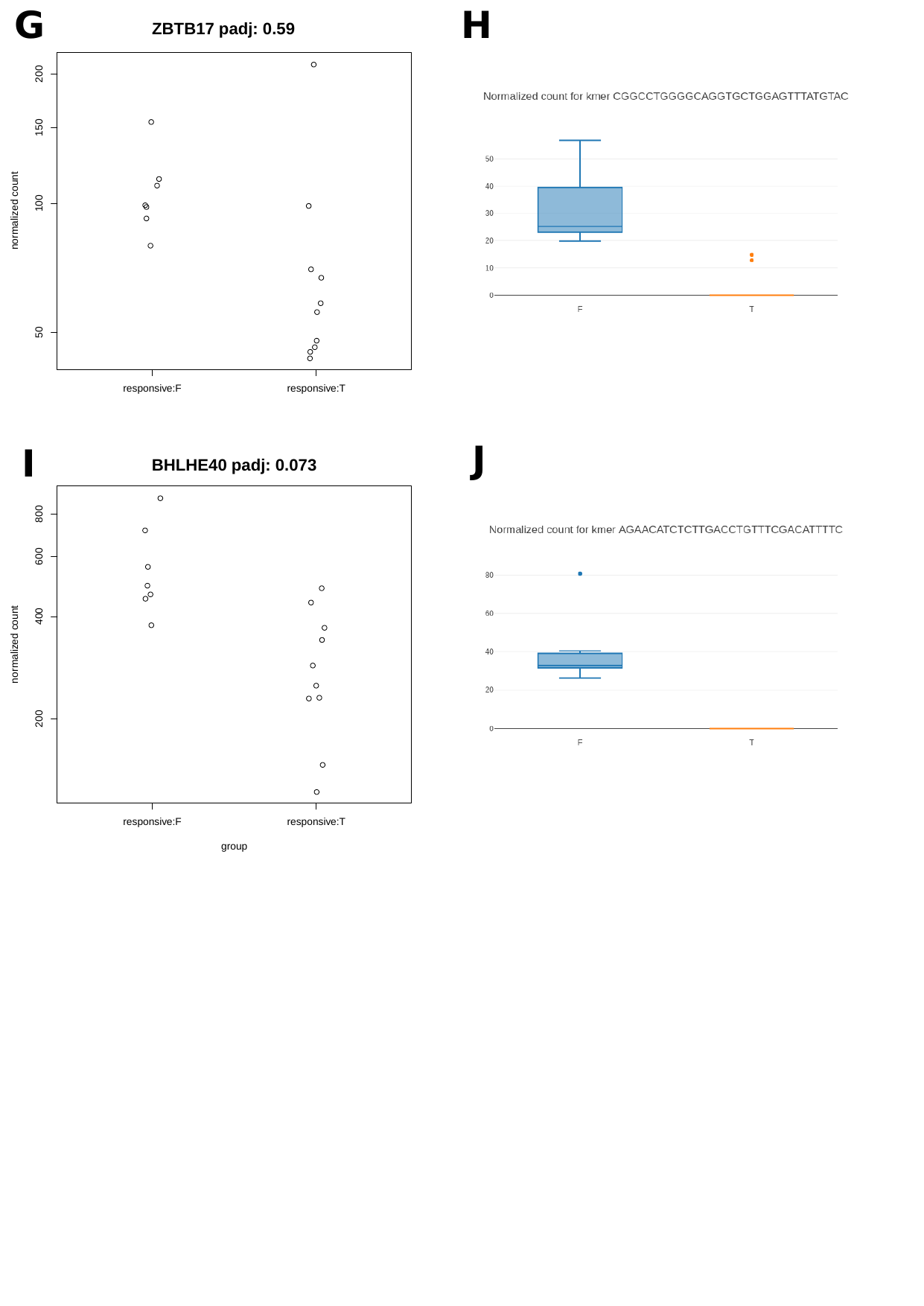
**Supplementary Figure 6:** DESEq2 normalized gene counts and the respective 𝑘-mer normalized abundances identified by iMOKAfor the genes ZBTB45 (**A-B**), AKT1 (**C-D**), BTBD9 (**E-F**), ZBTB17 (**G-H**) and BHLHE40 (**I-J**). The genes are not detected as differentially expressed using DESeq2 ( adjusted p-value on the top of each boxplot).


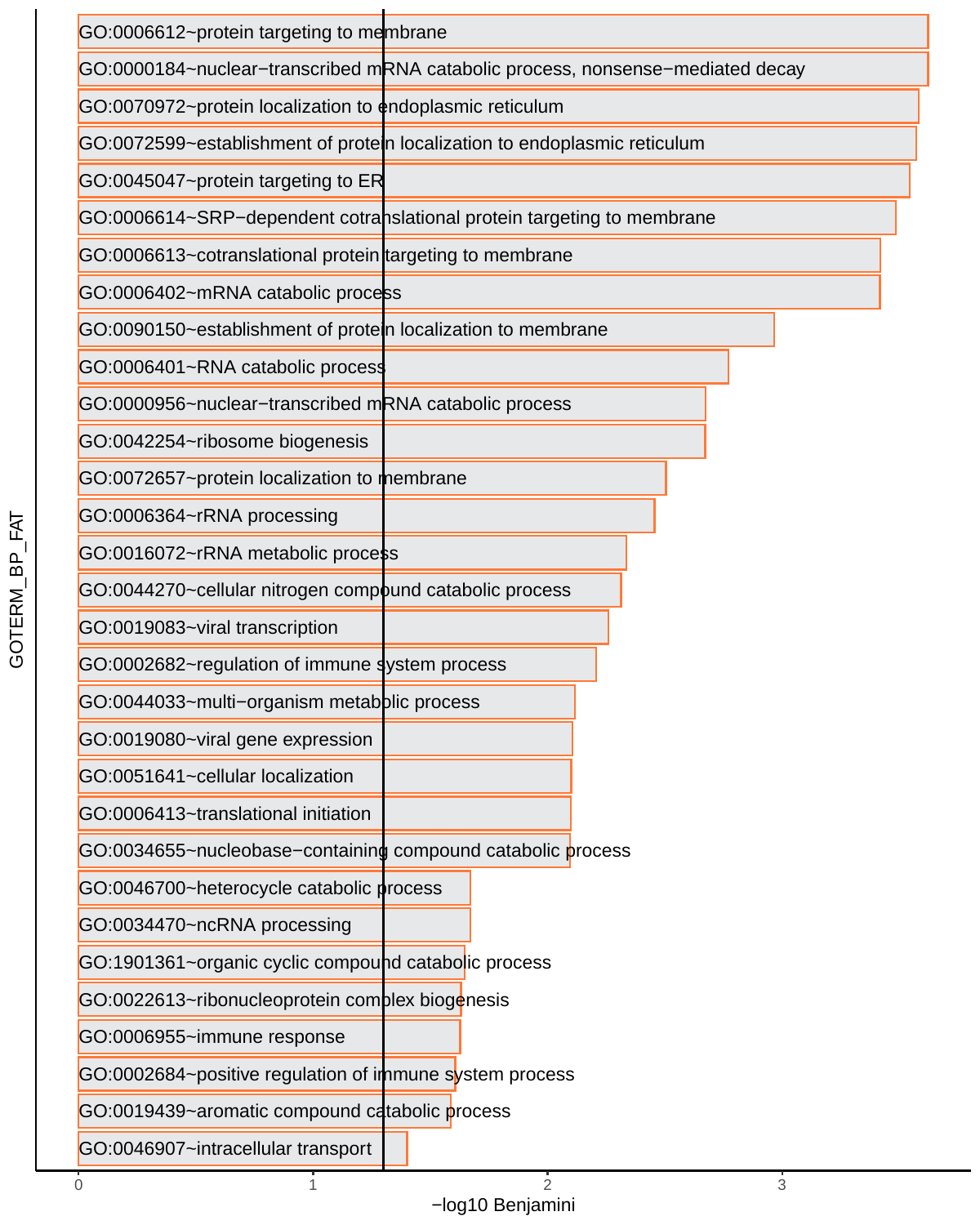


**Supplementary Figure 7:**Biological process gene ontology of the genes overlapped by the 𝑘-mers found by iMOKA in DLBCL.

**Supplementary tables**

| **Study** | **Event** | **Number of 𝑘-mers** |
| --- | --- | --- |
| TCGA_BC 3002 𝑘-mers | Gene | 742 |
|  | DE | 580 |
|  | unmapped | 523 |
|  | splice_borders | 479 |
|  | intergenic | 427 |
|  | intron | 162 |
|  | multiple_splice_junctions | 120 |
|  | mutation_borders | 86 |
|  | insertion_borders | 35 |
|  | deletion_borders | 12 |
|  | misalign | 1 |
| TCGA_OV  138 𝑘-mers | gene | 105 |
|  | unmapped | 14 |
|  | mutation_borders | 11 |
|  | intergenic | 8 |
| BEAUTY  1248 𝑘-mers | gene | 659 |
|  | DE | 20 |
|  | unmapped | 138 |
|  | splice_borders | 13 |
|  | intergenic | 377 |
|  | Intron | 25 |
|  | multiple_splice_junctions | 1 |
|  | mutation_borders | 13 |
|  | insertion_borders | 5 |
| DLBCL 1915 𝑘-mers | splice_borders | 588 |
|  | DE | 429 |
|  | intron | 74 |
|  | multiple_splice_junction | 12 |
|  | gene | 688 |
|  | unmapped | 168 |
|  | intergenic | 3 |
|  | multimap | 108 |

**Supplementary Table 1:** Number of 𝑘-mers found in each study and the genetic events (one 𝑘-mer can have multiple events) they are associated to. The results are not additionally filtered, an interactive view of the -mers is available using the supplementary data and iMOKA GUI. A detailed description of the events can be found in Supplementary Table 2.

| **Event name** | **Condition** | **Description** |
| --- | --- | --- |
| insertion_borders deletion_borders mutation_borders | 𝑘-mer overlaps an insertion/deletion/mutation and has a higher accuracy score than other 𝑘-mers within the same kmer graph. | This event overlaps a known variation event. |
| splice_borders | A splicing site where the overlapping 𝑘-mers have a higher accuracy than the others. | This event might overlap with a splicing site involved with alternative splicing derived isoforms or intron retention events. |
| DE | An annotated transcript is covered by filtered 𝑘-mers across more than 50% of its length (by default). | Generally a differentially expressed transcript. |
| Intron | An annotated intron is covered by filtered 𝑘-mers across more than 50% of its length (by default). | This event might overlap with a retained intron, an alternative starting site or an intronic transcript. |
| multiple_splice_junction | This event is called when there are two splicing sites with one common acceptor or donor site. | This splicing site can indicate two differentially expressed transcripts or a different transcript usage. |
| Intergenic | This event represents a sequence that mapped correctly but doesn’t overlap with any feature of the given annotation file. | These events might represent novel or unannotated transcripts. |
| gene | This event is generic and represents 𝑘-mers that map on a gene without any other event associated with it. | This event is generally associated with very small transcripts or overlapping genes. |
| unmapped | Sequences that didn't map on the given reference genome. | These can be due to contamination, repetitive elements excluded by the aligner or chimeric transcripts. |
| multimap | Sequences that mapped to multiple positions of the reference genome with the same score | These can be repetitive elements, portions of pseudogenes or similar cases. |
| misalign | The sequence generated by the graph was mapped correctly, but the best 𝑘-mer is completely in the clipped region. | Those events should be rare and require a manual investigation. |

**Supplementary Table 2:** Description of the events in which iMOKA categorize the group of 𝑘-mers during the aggregation step.

**Supplementary references:**

1. Thorvaldsdóttir, H., Robinson, J. T. & Mesirov, J. P. Integrative Genomics Viewer (IGV): high-performance genomics data visualization and exploration. *Brief. Bioinform.* **14**, 178–192 (2013).

2. Plotly, T. Plotly: Collaborative data science. https://plot.ly. https://plot.ly (2015).

3. *Electron- https://www.electronjs.org/*.

4. *Angular - https://angular.io/*.

5. Yoo, A. B., Jette, M. A. & Grondona, M. SLURM: Simple Linux Utility for Resource Management. in *Job Scheduling Strategies for Parallel Processing* (eds. Feitelson, D., Rudolph, L. & Schwiegelshohn, U.) 44–60 (Springer, 2003). doi:10.1007/10968987_3.

6. Patro, R., Duggal, G., Love, M. I., Irizarry, R. A. & Kingsford, C. Salmon provides fast and bias-aware quantification of transcript expression. *Nat. Methods* **14**, 417–419 (2017).

7. Bjørklund, S. S. *et al.* Widespread alternative exon usage in clinically distinct subtypes of Invasive Ductal Carcinoma. *Sci. Rep.* **7**, 5568 (2017).
