# Supplementary figures and images for: iMOKA: k-mer based software to analyze large collections of sequencing data
Source: Genome Biol. 2020 Oct 13;21:261. doi: 10.1186/s13059-020-02165-2 (PMC7552494; doi:10.1186/s13059-020-02165-2)

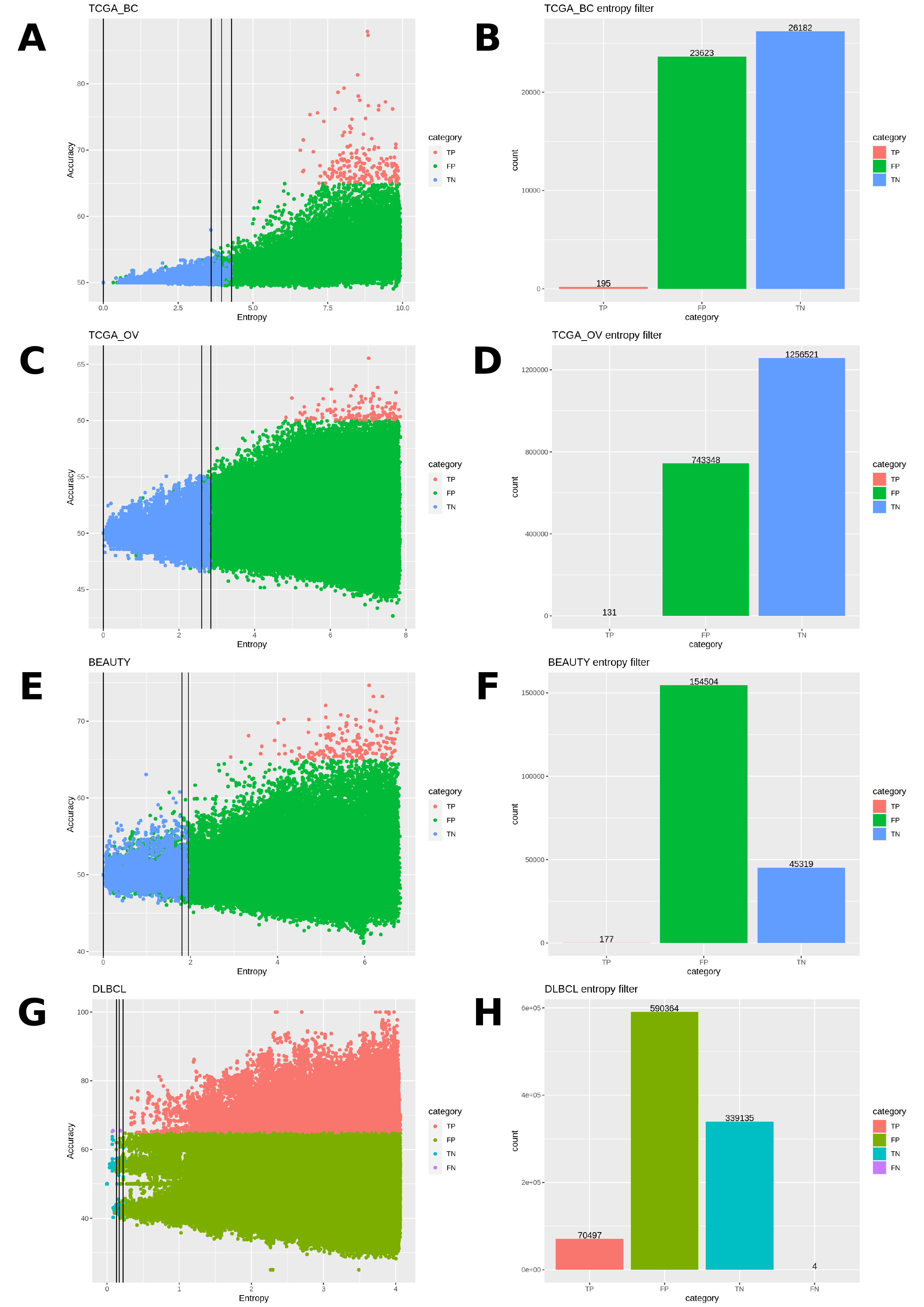

Supplement: Supplementary file 7 — Additional file 7. Supplementary Figures S1-S7. [file 13059_2020_2165_MOESM7_ESM.zip › Figure_S1.png]

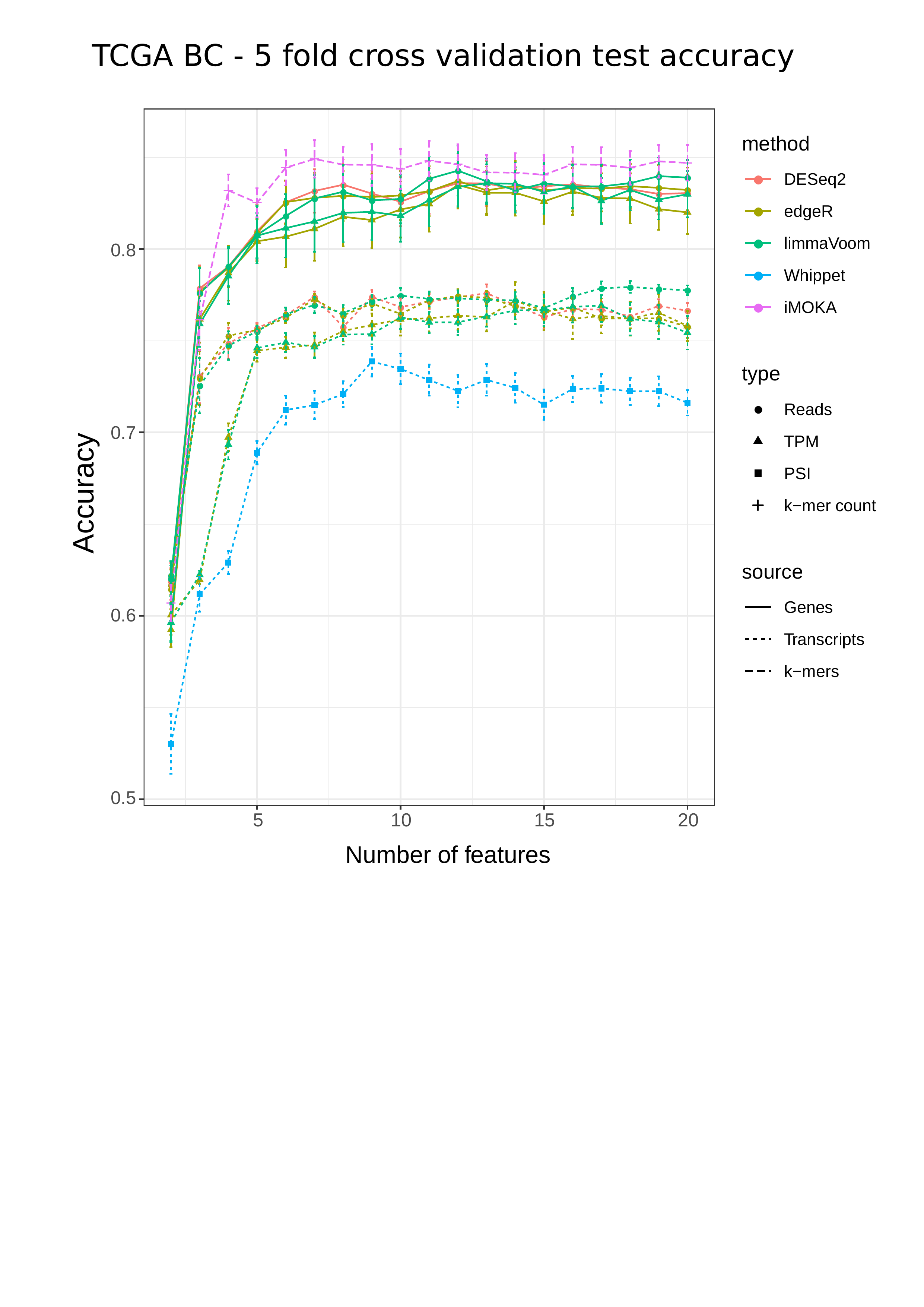

Supplement: Supplementary file 7 — Additional file 7. Supplementary Figures S1-S7. [file 13059_2020_2165_MOESM7_ESM.zip › Figure_S2.png]

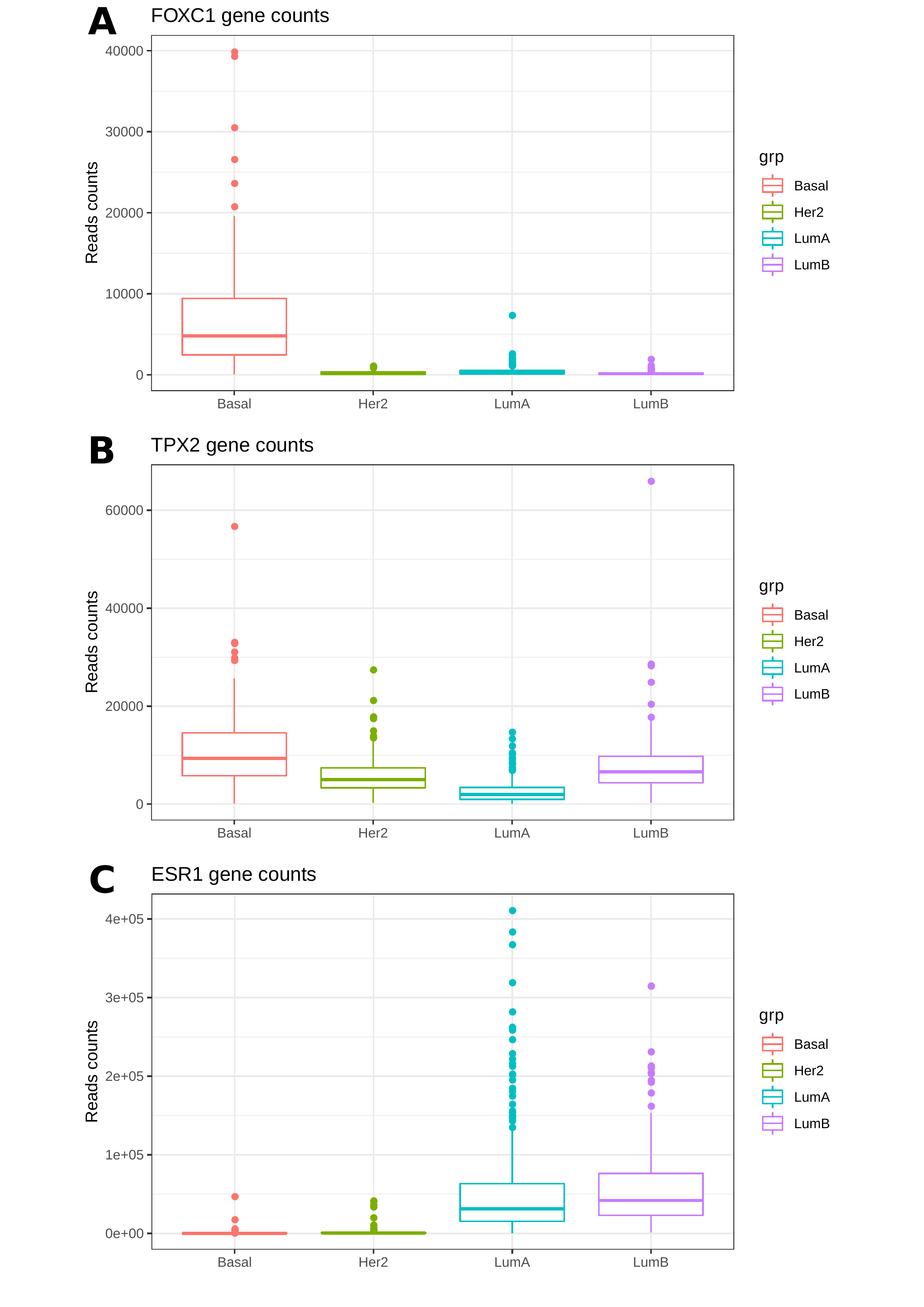

Supplement: Supplementary file 7 — Additional file 7. Supplementary Figures S1-S7. [file 13059_2020_2165_MOESM7_ESM.zip › Figure_S3.png]

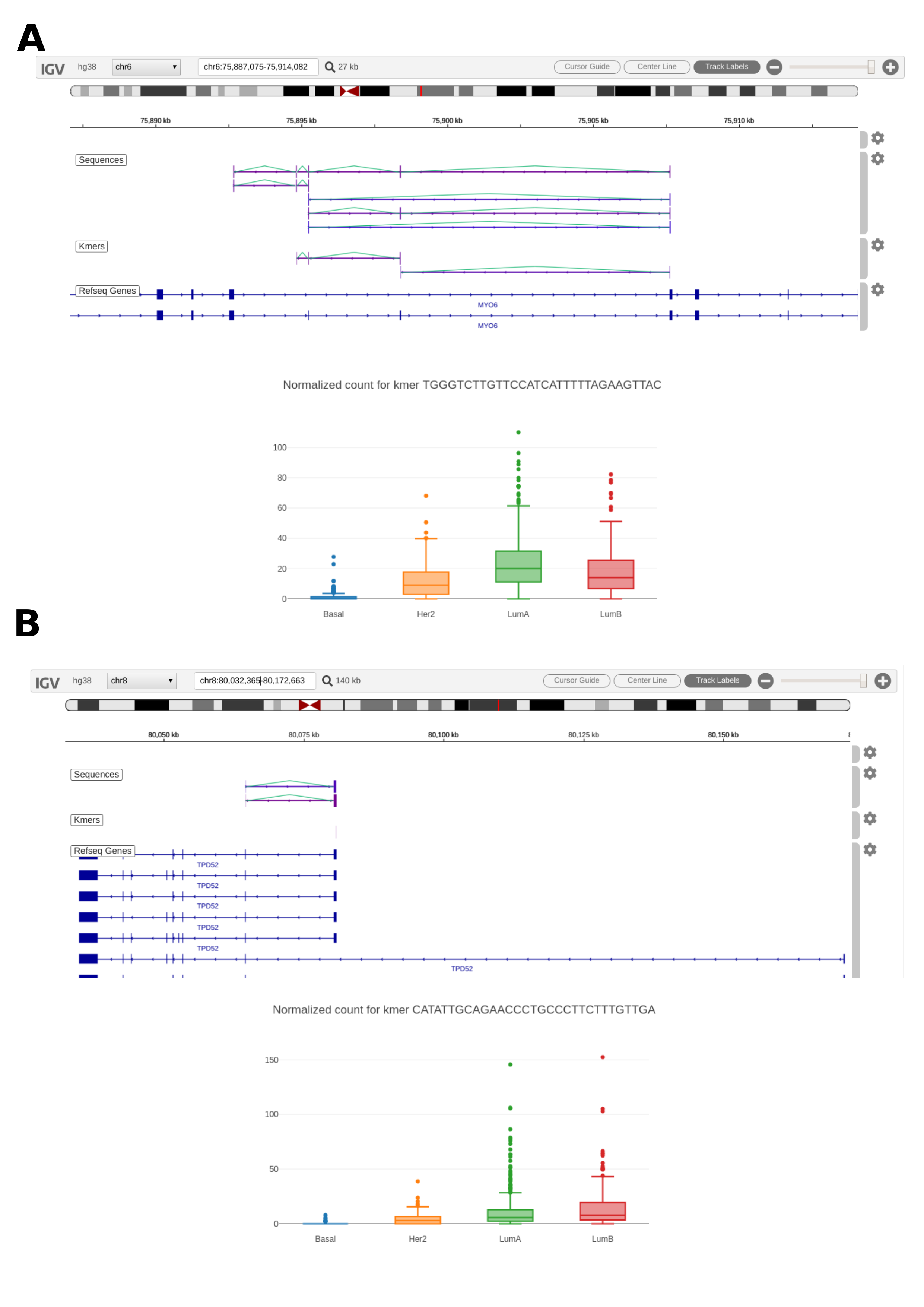

Supplement: Supplementary file 7 — Additional file 7. Supplementary Figures S1-S7. [file 13059_2020_2165_MOESM7_ESM.zip › Figure_S4a.png]

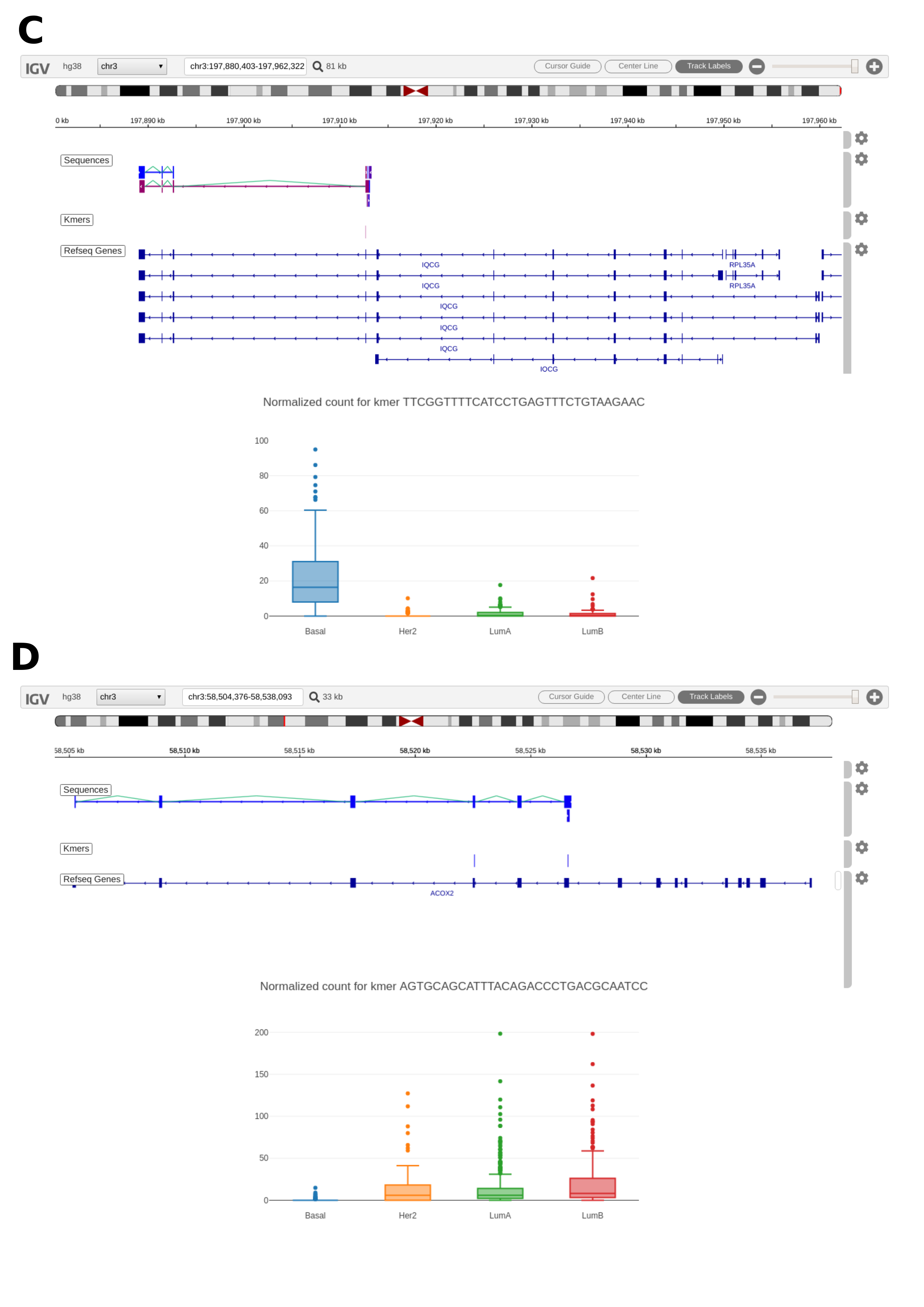

Supplement: Supplementary file 7 — Additional file 7. Supplementary Figures S1-S7. [file 13059_2020_2165_MOESM7_ESM.zip › Figure_S4b.png]

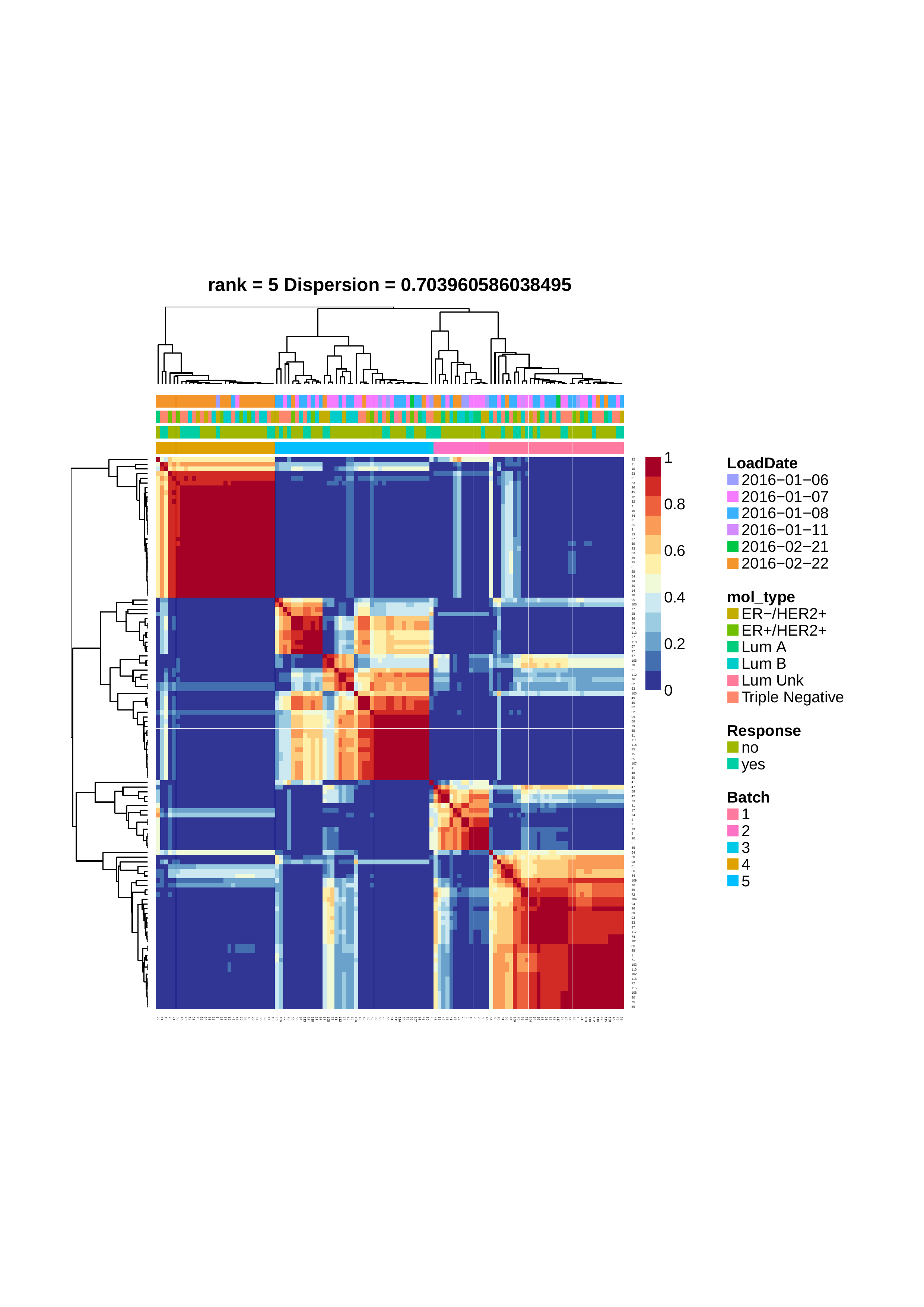

Supplement: Supplementary file 7 — Additional file 7. Supplementary Figures S1-S7. [file 13059_2020_2165_MOESM7_ESM.zip › Figure_S5.png]

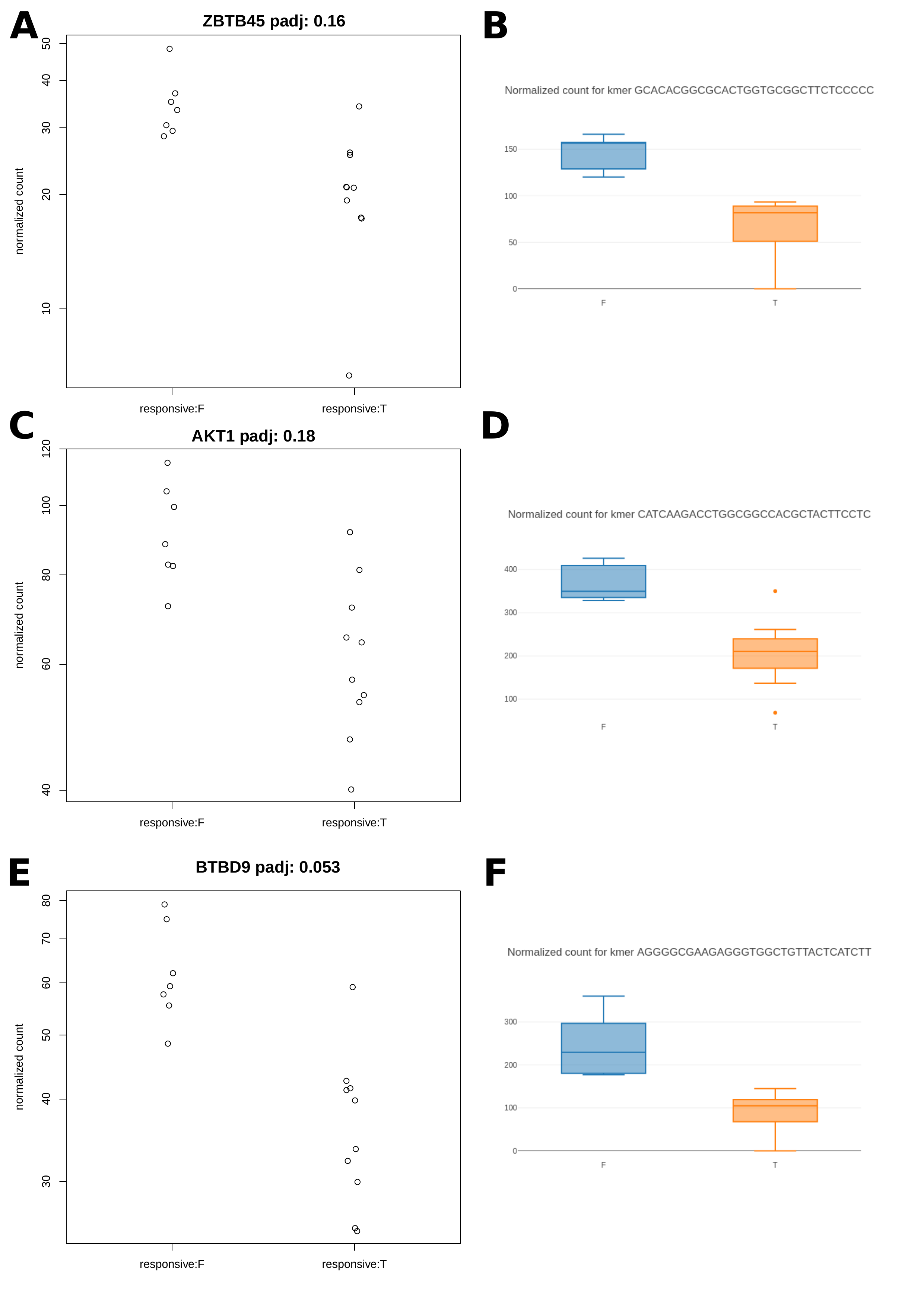

Supplement: Supplementary file 7 — Additional file 7. Supplementary Figures S1-S7. [file 13059_2020_2165_MOESM7_ESM.zip › Figure_S6a.png]

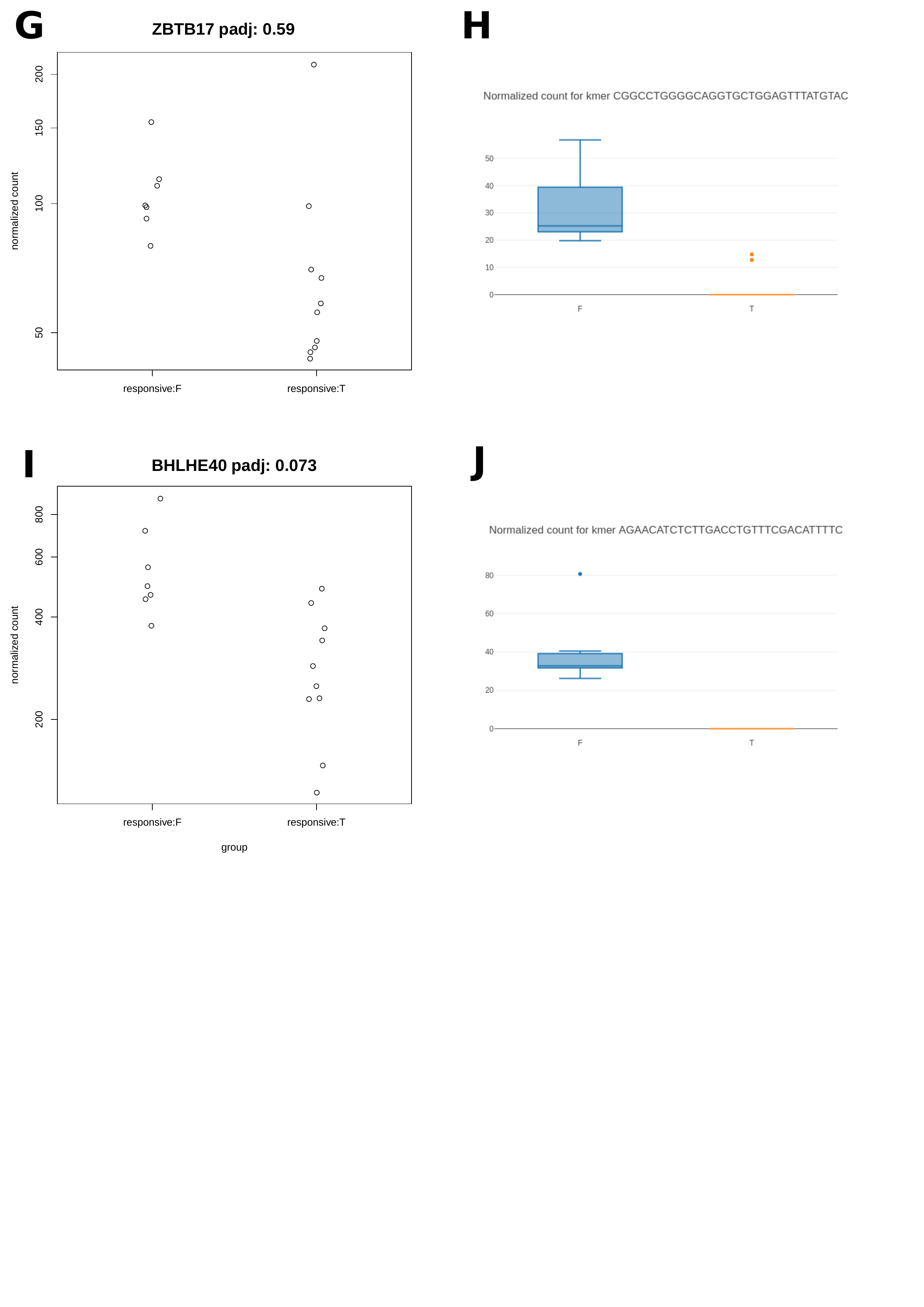

Supplement: Supplementary file 7 — Additional file 7. Supplementary Figures S1-S7. [file 13059_2020_2165_MOESM7_ESM.zip › Figure_S6b.png]

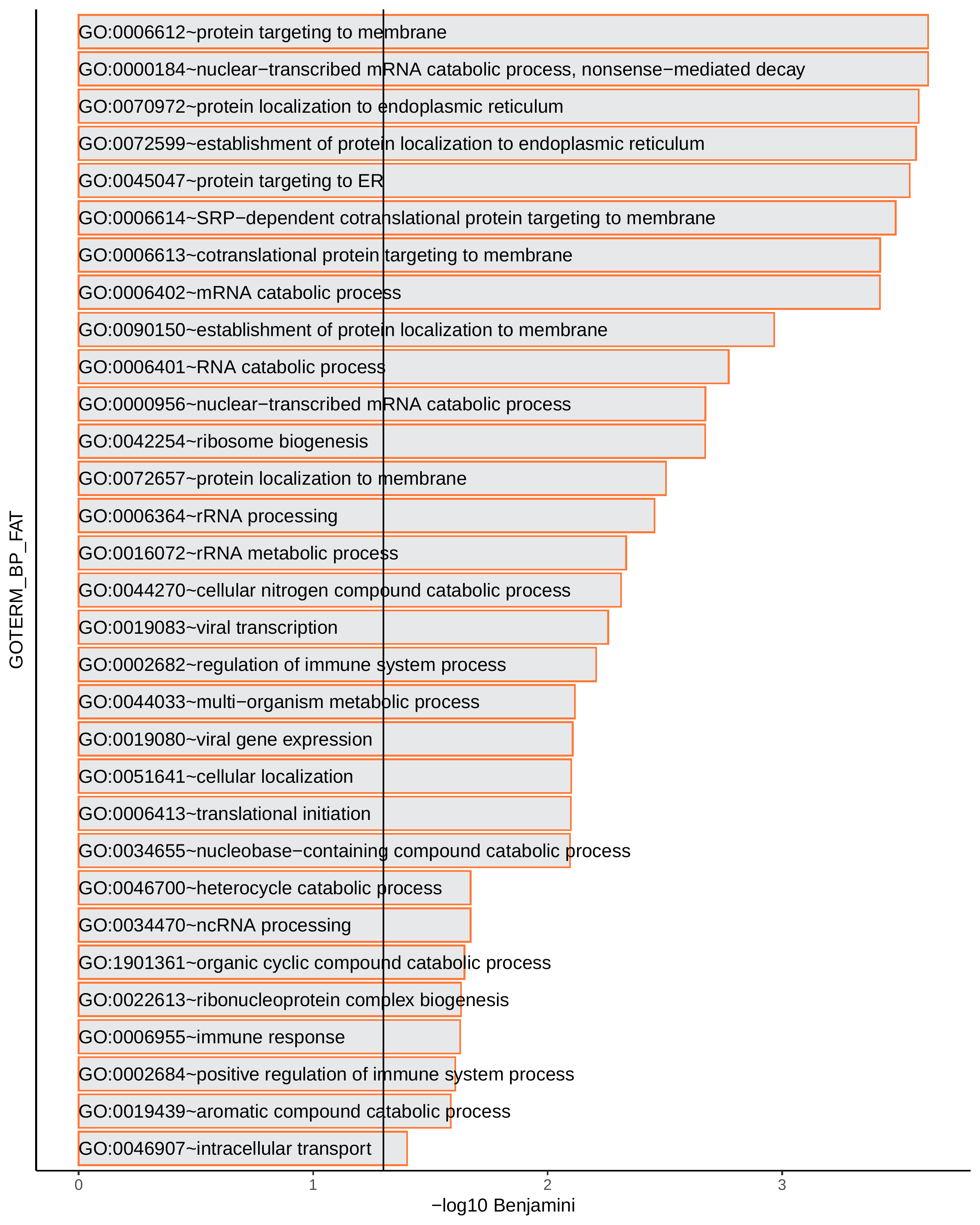

Supplement: Supplementary file 7 — Additional file 7. Supplementary Figures S1-S7. [file 13059_2020_2165_MOESM7_ESM.zip › Figure_S7.png]
