# Supplementary material for: iMOKA: k-mer based software to analyze large collections of sequencing data
Source: Genome Biol. 2020 Oct 13;21:261. doi: 10.1186/s13059-020-02165-2 (PMC7552494; doi:10.1186/s13059-020-02165-2)
Supplement: Supplementary file 8 — Additional file 8. Review history. [file 13059_2020_2165_MOESM8_ESM.docx]

Review History

**First round of review**

**Reviewer 1**

**Were you able to assess all statistics in the manuscript, including the appropriateness of statistical tests used?**

Yes: NA

**Were you able to directly test the methods?**

No

**Comments to author:**

Major Revisions:
-     Breast and ovarian cancer are both predominantly hormone driven and female only cancers. When reviewing k-mers there are prominent differences between male and female. In order to accurately exhibit the application of iMOKA as described in the manuscript, the authors need to demonstrate the use of their software in mixed-gender studies.

-     Line 73 - The authors state that they employ a "Monte Carlo cross validation with a high number of iterations and an early break that efficiently reduces overfitting and generates predictions that overcome batch effects". It would be beneficial to the quality of the manuscript if the authors demonstrated the performance of this approach in data with prominent batch effects.

Questions:
-     Upon attempting to build the singularity I received an error and was subsequently unable to evaluate the software that the manuscript describes. The error I received informed me that I was unauthorised to access the resource. I presume that the authors intend to make the software available in the near future?


Minor Revisions:
-     There is a mild inconsistency regarding input file formats. Line 60 describes the input file formats to be: "FASTQ, FASTA, BAM format or SRR". Line 89 reflects these file formats. Line 251 mentions input file formats but does not mention FASTA.

-     Supplementary figure S1 legend. The section reads 'having entropy slower than 0.5 contains'. Presumably the text is supposed to read 'lower' rather than 'slower'. Additionally, the following 'Entropy' does not require a capital letter.

-     I found the equation on line 283 hard to follow and would benefit from additional clarity.

-     Although it is not ubiquitous throughout the literature, the k in k-mer should be italicised throughout the document as it pertains to a substring of length k, as the authors duly note on line 39. Particularly on line 61 where the authors directly refer to a "mer of given length k"

-     It is common convention to italicise gene names (such as on lines 134-136). Although I am unsure if this is a requirement of this journal.

-     Line 264 - The base of a log function should be subscript ie log2 should be log2 as in the equation on line 345.

-     The "Where" on line 309 should have a colon after it in keeping with the rest of the manuscript.

-     I am not sure if there are figures or equations linked with Algorithm 1. If so, I have been unable to locate them.

-     Figure 1 would benefit from the additional mention of the entropy filtering step.

-     As the first mention of the adjustment parameters, I found line 353 somewhat difficult to follow. I think this line would benefit from some clarification.

Additional Suggestions:
-     The initial software build command uses sudo. If possible, I would suggest the authors to pre-build the singularity image file and host the file on sylabs/docker so that the software can simply be installed with the 'singularity pull' command. This would increase the usability of the software and prevent the requirement for system administrators to install the software on behalf of the user.

-     It would be interesting to observe the performance in mixed-technology analysis (such as whole genome / RNA sequencing) in addition to the batch effect.


-     Line 118: "To reduce overfitting, we used a Monte-Carlo cross validation approach and down sampled the largest classes to create balanced sized classes." In datasets with extremely unbalanced classes, down sampling risks discarding a large amount of potentially useful information. Have the authors attempted up-sampling the minority class using a technique such as SMOTE (synthetic minority over-sampling technique) or otherwise? Additionally, are there protections built into iMOKA to limit users misinterpreting results (for example in minority groups consisting of too few samples)?

-     Sometimes pre-processing of sequence data results in single ended files in addition to the processed pair. I wonder, is there a way in iMOKA to include multiple sequencing files per sample? Ie 2x paired end file and 1x single end file? I feel the approach may gain sensitivity by allowing more input files per sample.

**Reviewer 2**

**Were you able to assess all statistics in the manuscript, including the appropriateness of statistical tests used?**

Yes: details in the comments

**Were you able to directly test the methods?**

No

**Comments to author:**

This study presents a new software, iMOKA, for utilizing k-mers for sequencing data analysis. Through three real data applications, the authors show that the k-mers processed from iMOKA can be used as features in prediction problems; these prediction problems include classification of cancer subtypes and classification of response to chemotherapy. In addition, the authors showed a few examples where some of the selected k-mers from iMOKA can be used for inferring SNPs and gene isoform usage. It is an interesting idea to make use of k-mers for sequencing data analysis. However, I have two major concerns. First, a key disadvantage of iMOKA is that it requires knowing sample group assignment a priori. Requires knowing the sample group assignment, such as the cancer subtypes and response to chemotherapy, prior to predicting such group assignment, gives iMOKA an unfair advantage over the other existing feature extraction and prediction methods in all the comparisons carried out in the present study. In addition, the real data applications in the paper are not yet well developed: the paper currently lacks an unbiased way to assess the biological relevance of the features identified from iMOKA in all three data applications; and it is unclear what novel findings can iMOKA provide other than reproducing findings from previous studies. Specifically:  

1. Two k-mer filtering steps, the naive Bayes filter and Shannon entropy, are employed in iMOKA for selecting k-mers. Both these two filtering steps, however, require knowing the sample group assignment. That is, iMOKA needs to know which of the four cancer types each sample belongs to in the first data and whether the sample underwent the chemo treatment the second and third data before making any predictions. Based on what is described in the paper, all samples along with their group assignments are currently used for both these two filtering steps. If this is the case, then these two filtering steps effectively extracted k-mer features using the group information on all samples which are further used in the later random forest classifier for classifying samples; thus leading to an unfair advantage for iMOKA over the other compared methods in all the comparisons. Since features are extracted knowing the sample group information, they are expected to yield better performance in the later steps for predicting the same sample group assignments. To ensure a fair comparison among methods, the authors should divide the data into a training set and a test set at the beginning. Both Naive Bayes classifier and Shannon entropy should be computed in the training data set only (and for the naive Bayes classifier, you can still divide the training set into training and test sets further). The selected k-mers used in the random forest classifier should not have access to the sample group information in the test set.

2. For the random forest classifier, "for each method considered, a decision tree classifier is trained with all the samples". This is also not a proper way for performing cross-validation. The authors should perform feature selection using the decision tree classifier in the training data only. Otherwise, some of the compared methods that have less features than iMOKA to begin with may be penalized during the comparison.

3. The results are not well developed. Currently it lacks an unbiased way of assessing the biological relevance of the features identified from iMOKA in all three data applications. Many presented results appear to be cherry picking. For example, in the first data, "iMOKA found genes that have been extensively linked to breast cancer subtypes and are already used in the clinic." However, it is not reported what is the proportion of genes identified by iMOKA that have been linked previously to breast cancer subtypes, and what's the proportion of genes identified by iMOKA that have not been linked to previous studies and thus may be potential false signals. In the second data,  "iMOKA detected a known nonsense mutation (SNP id: rs10794537) in the alpha-L-iduronidase (IDUA) gene.", but it is unclear how many nonsense mutations in total are identified by iMOKA and what is the proportion of them that have previous literature support. In the third data, iMOKA "can identify multiple events on the same gene that are useful for classification", but it is unclear how many genes have such events, what are these genes, and how many of them are related to cancer. In addition, because these examples all point to findings that are well known in the cancer literature, it is also unclear at the moment what novel findings can be achieved by iMOKA.

**Reviewer 3**

**Were you able to assess all statistics in the manuscript, including the appropriateness of statistical tests used?**

Yes: I have checked all statistics in the manuscript and methods are generally sounded. Some clarifications are asked to the authors in the review.

**Were you able to directly test the methods?**

No

**Comments to author:**

Deepening in their research on the use of k-mers for genomics data analysis, Lorenzi and colleagues have developed and implemented a new computational method called iMOKA that improves previous algorithms in its computational efficiency and accuracy. In my opinion, iMOKA represents a major improvement in the way genomics data might be analyzed. As clearly presented in datasets 2 and 3, its power lies in the integrative use of the data. If SNVs, gene expression or isoform usage, iMOKA is able to identify the best predictors in different biological setups without the need to explore all the sequences generated in a NGS experiment but only those that seem to be the best candidates. Also, as in dataset 3, there might be different features of a gene that can be relevant to a given disease. iMOKA also helps identifying the potential mechanisms of action of given genomic features, beyond the use of different technologies and data analysis pipelines.

There are only a few clarifications on the methodological decisions made, that should be done prior manuscript publication:
Methods:
*     How did you come up with RF=10^9?
*     I think the idea of the entropy filter is really great, to remove k-mers irrelevant for the classification of the samples. However, it is not totally clear to me how you update the threshold. Isnt it dangerous that it is adaptive? Couldn't you be removing relevant kmers as you go further in the process?
*     Once more, there is not a convincing explanation of how to set the threshold for the overlapping of the k-mers in the graph
*     Graph mapping and annotation:
o     alternative splice sites are identified when a k-mer graph is split across exons. How efficient is that? How do you do it? Coordinates comparison?
o     differential expression (DE) is identified if 50% (set by parameter d) of an annotated transcript  is covered by the k-mer graphs. Why 50%? Could you please clarify that?
o     Unmapped or multimapped events. How many of these do you get in average? Don't you consider a probabilistic approach (EM like) to identify the most likely genomic location that generated the read? How do you interpret those that are not annotated?
Results:
*     Figure 2C. Would be nice to compare against plain gene quantification, probably these genes are very predictive anyway (i.e. ESR1)
*     The OV cancer example is really nice. I agree that the gain might be due to the fact that the most discriminative k-mers are point mutations. Also the third example is really nice and shows the true potential of iMOKA

**Authors Response**

**Point-by-point responses to the reviewers’ comments:**

Comment

*Response*

*We thank the reviewers for reviewing our manuscript. Globally we agree with their comments and have tried our best to strengthen the weak points that they pointed out.

Briefly, the major additions to our manuscript include:
-A study with both male and female subjects
-An analysis of batch effects in the studies we used
-A test set to evaluate the entire iMOKA pipeline
-The use of an out of bag metric to evaluate the RF models
-Gene ontology enrichment for each experiment
-An analysis of the performance of the Entropy filter
-Clarifications and guidelines regarding multiple options*

*Below we include a point by point response to the reviewers concerns.*

Reviewer #1: Major Revisions:
- Breast and ovarian cancer are both predominantly hormone driven and female only cancers. When reviewing 𝑘-mers there are prominent differences between male and female. In order to accurately exhibit the application of iMOKA as described in the manuscript, the authors need to demonstrate the use of their software in mixed-gender studies.
*If we understand correctly, reviewer #1 is concerned that sex bias may be a major hurdle for classification using 𝑘-mer counts. Of course this is true in many studies and approaches and is the reason why gender balance as well as numerous other confounding factors are generally taken into account during study design.
However, the reviewer raises a valid point regarding sex specifically because the presence of a different chromosome, Y, generates different 𝑘-mers, increasing the 𝑘-mer heterogeneity only of the males. To investigate this potential bias, we performed two experiments.
Firstly, as requested by the reviewer #1 we included a mixed-gender study of target RNA-seq: the DLBCL study. We performed end-to-end iMOKA analysis and again found that iMOKA performed extremely well (Figure 5).
Secondly in order to directly verify whether the sex chromosomes affect iMOKA 𝑘-mer count normalization we produced a matrix where we removed 𝑘-mers from the sex chromosomes from each patient’s bam file. We found that the counts of the common 𝑘-mers between the full matrix and the “asexual” matrix have a correlation coefficient >= 0.99 for the 98,9% of the 𝑘-mers. Although sex biases can of course arise from other regions of the genome, we were reassured to see that removing the X and Y chromosomes had close to no effect on the 𝑘-mer counts of the other 𝑘-mers.*


- Line 73 - The authors state that they employ a "Monte Carlo cross validation with a high number of iterations and an early break that efficiently reduces overfitting and generates predictions that overcome batch effects". It would be beneficial to the quality of the manuscript if the authors demonstrated the performance of this approach in data with prominent batch effects.
*Again, we agree. To this end we used a published software dedicated to finding batch effects called DASC (Ref 31 of the main text). We were clearly able to identify a batch effect of the BEAUTY dataset (Figure S5). This effect was associated with the date of upload of a specific batch. This has been integrated into the manuscript.
For the other data sets, although DASC also found batch effects, we lacked sufficient metadata to associate them with a specific bias.*


Questions:
- Upon attempting to build the singularity I received an error and was subsequently unable to evaluate the software that the manuscript describes. The error I received informed me that I was unauthorised to access the resource. I presume that the authors intend to make the software available in the near future?
*Apologies for this: when we moved the github repository from private to public, we forgot to do the same for the docker hub repository. The problem is now fixed.
The singularity images ( basic and the extended, that includes the BLAT hg38 reference genome and GENCODE v29 human annotations ) are pre-built in the github release section https://github.com/RitchieLabIGH/iMOKA/releases
under the “assets” files, alongside with the GUI release for different architectures. They are always updated to the latest version (since we submitted, we updated essentially graphical bugfix, nothing concerning the algorithm itself). Since the access to this page was not clear, we have now modified the README files of the github to make this clearer and of easier access.*

Minor Revisions:
- There is a mild inconsistency regarding input file formats. Line 60 describes the input file formats to be: "FASTQ, FASTA, BAM format or SRR". Line 89 reflects these file formats. Line 251 mentions input file formats but does not mention FASTA.
*Corrected*

- Supplementary figure S1 legend. The section reads 'having entropy slower than 0.5 contains'. Presumably the text is supposed to read 'lower' rather than 'slower'. Additionally, the following 'Entropy' does not require a capital letter.
*Corrected*

- I found the equation on line 283 hard to follow and would benefit from additional clarity.
*We included an example and simplified the notation*

- Although it is not ubiquitous throughout the literature, the k in 𝑘-mer should be italicised throughout the document as it pertains to a substring of length k, as the authors duly note on line 39. Particularly on line 61 where the authors directly refer to a "mer of given length k"
*Corrected*

- It is common convention to italicise gene names (such as on lines 134-136). Although I am unsure if this is a requirement of this journal.
*We didn’t find any indication in the guidelines referring to this topic, we’ll ask to the editor.*


- Line 264 - The base of a log function should be subscript ie log2 should be log2 as in the equation on line 345.
*Corrected*


- The "Where" on line 309 should have a colon after it in keeping with the rest of the manuscript.
*Corrected*


- I am not sure if there are figures or equations linked with Algorithm 1. If so, I have been unable to locate them.
*The image was not present in the text for mistake and we clarified the procedure in the text to avoid the presence of additional figures.*

- Figure 1 would benefit from the additional mention of the entropy filtering step.
*The entropy filter is used to reduce the number of 𝑘-mers analysed and is totally dependent on the Bayesian classifier’s results. We think it might be misleading to include it in the graphical representation, since it’s more related to the implementation optimization rather than the algorithm itself. We included it in the description of the figure and we added a clarification about the actual role of the entropy filter in the main text.*


- As the first mention of the adjustment parameters, I found line 353 somewhat difficult to follow. I think this line would benefit from some clarification
*done*

Additional Suggestions:
- The initial software build command uses sudo. If possible, I would suggest the authors to pre-build the singularity image file and host the file on sylabs/docker so that the software can simply be installed with the 'singularity pull' command. This would increase the usability of the software and prevent the requirement for system administrators to install the software on behalf of the user.
*As mentioned above, the compiled singularity images are available in the github release page and the README file had been modified in order to better guide the user.*

- It would be interesting to observe the performance in mixed-technology analysis (such as whole genome / RNA sequencing) in addition to the batch effect.
*We absolutely agree that it would be interesting and has been a subject of interest of ours. We have two hurdles that we are currently trying to overcome. Firstly, we are having trouble locating perfectly matched RNA and whole genome data across a large number of well annotated patients. The second is that we anticipate these two types of data to have very different behaviours (changes in RNA are probably more quantitative than nucleotide variations found in genomes). Should we mix the 𝑘-mers from both or should we keep track of which 𝑘-mer comes from which type of sequencing? These are issues we are currently investigating.*


- Line 118: "To reduce overfitting, we used a Monte-Carlo cross validation approach and down sampled the largest classes to create balanced sized classes." In datasets with extremely unbalanced classes, down sampling risks discarding a large amount of potentially useful information. Have the authors attempted up-sampling the minority class using a technique such as SMOTE (synthetic minority over-sampling technique) or otherwise? Additionally, are there protections built into iMOKA to limit users misinterpreting results (for example in minority groups consisting of too few samples)?
*Again, this is something we discussed extensively. Techniques of over-sampling are very useful but come with drawbacks: the newly generated samples are produced using the shapes of the minority group, without adding new information and this would drive the machine learning process to give more weight to the information contained in the over-sampled group.
The Monte-Carlo cross validation with a high number of iterations reduces the loss of useful information increasing the combinations of training/test group generated, at the cost of a higher computational time.
Finally, there are no protections built into iMOKA to limit the number of samples in each group because this depends a lot by the type of data and the complexity of the discrimination of the two groups, the presence of outliers or misclassified samples. Following the suggestion of the reviewer #1, we have added guidelines in the github README file and in the GUI.*


- Sometimes pre-processing of sequence data results in single ended files in addition to the processed pair. I wonder, is there a way in iMOKA to include multiple sequencing files per sample? Ie 2x paired end file and 1x single end file? I feel the approach may gain sensitivity by allowing more input files per sample.
*Currently there is no limit in the number of files that iMOKA can process for each sample, it’s sufficient to add them in the last column of the preprocessing input file and separate them by semicolons (;) , as for paired end.*


Reviewer #2: This study presents a new software, iMOKA, for utilizing 𝑘-mers for sequencing data analysis. Through three real data applications, the authors show that the 𝑘-mers processed from iMOKA can be used as features in prediction problems; these prediction problems include classification of cancer subtypes and classification of response to chemotherapy. In addition, the authors showed a few examples where some of the selected 𝑘-mers from iMOKA can be used for inferring SNPs and gene isoform usage. It is an interesting idea to make use of 𝑘-mers for sequencing data analysis. However, I have two major concerns. First, a key disadvantage of iMOKA is that it requires knowing sample group assignment a priori. Requires knowing the sample group assignment, such as the cancer subtypes and response to chemotherapy, prior to predicting such group assignment, gives iMOKA an unfair advantage over the other existing feature extraction and prediction methods in all the comparisons carried out in the present study. In addition, the real data applications in the paper are not yet well developed: the paper currently lacks an unbiased way to assess the biological relevance of the features identified from iMOKA in all three data applications; and it is unclear what novel findings can iMOKA provide other than reproducing findings from previous studies. Specifically:

1. Two 𝑘-mer filtering steps, the naive Bayes filter and Shannon entropy, are employed in iMOKA for selecting 𝑘-mers. Both these two filtering steps, however, require knowing the sample group assignment. That is, iMOKA needs to know which of the four cancer types each sample belongs to in the first data and whether the sample underwent the chemo treatment the second and third data before making any predictions. Based on what is described in the paper, all samples along with their group assignments are currently used for both these two filtering steps. If this is the case, then these two filtering steps effectively extracted 𝑘-mer features using the group information on all samples which are further used in the later random forest classifier for classifying samples; thus leading to an unfair advantage for iMOKA over the other compared methods in all the comparisons. Since features are extracted knowing the sample group information, they are expected to yield better performance in the later steps for predicting the same sample group assignments. To ensure a fair comparison among methods, the authors should divide the data into a training set and a test set at the beginning. Both Naive Bayes classifier and Shannon entropy should be computed in the training data set only (and for the naive Bayes classifier, you can still divide the training set into training and test sets further). The selected 𝑘-mers used in the random forest classifier should not have access to the sample group information in the test set.

 *We don’t entirely agree that iMOKA is given an unfair advantage as all the approaches we compare it to also make use of class labels and the entire set of samples to perform their differential representation step. The final output of all the three approaches is a subset of features that are different between the given groups of samples.
However we do entirely agree that from a ML methodology perspective the test set should never be exposed to the model while it is being created. Therefore, we sought to perform a 5 fold cross validation using 4/5 of the samples to reduce the features using each method and evaluated the accuracy on the remaining 1/5 (now a genuine test set). However, when doing this we reduce the number of samples available. For the TCGA Breast Cancer dataset
this is feasible as there are over 1000 samples and we now include this analysis in Supplementary Figure 2 where iMOKA still clearly outperforms the other approaches. However, for the other datasets because their sample numbers were already low, by removing a large portion of the samples at the start, none of the approaches are able to generate good models (<70% accuracy).*

2. For the random forest classifier, "for each method considered, a decision tree classifier is trained with all the samples". This is also not a proper way for performing cross-validation. The authors should perform feature selection using the decision tree classifier in the training data only. Otherwise, some of the compared methods that have less features than iMOKA to begin with may be penalized during the comparison.

*Agreed, we now use the out of bag error. In their paper entitled “out of bag error” Breiman et al., (OUT-OF-BAG ESTIMATION. in (1996)) give empirical evidence to show that the out-of-bag estimate is as accurate as using a test set of the same size as the training set and considered a go to method for evaluating RF models.*


3. The results are not well developed. Currently it lacks an unbiased way of assessing the biological relevance of the features identified from iMOKA in all three data applications. Many presented results appear to be cherry picking. For example, in the first data, "iMOKA found genes that have been extensively linked to breast cancer subtypes and are already used in the clinic." However, it is not reported what is the proportion of genes identified by iMOKA that have been linked previously to breast cancer subtypes, and what's the proportion of genes identified by iMOKA that have not been linked to previous studies and thus may be potential false signals. In the second data, "iMOKA detected a known nonsense mutation (SNP id: rs10794537) in the alpha-L-iduronidase (IDUA) gene.", but it is unclear how many nonsense mutations in total are identified by iMOKA and what is the proportion of them that have previous literature support. In the third data, iMOKA "can identify multiple events on the same gene that are useful for classification", but it is unclear how many genes have such events, what are these genes, and how many of them are related to cancer. In addition, because these examples all point to findings that are well known in the cancer literature, it is also unclear at the moment what novel findings can be achieved by iMOKA.

*We understand the reviewer's concern regarding cherry picking the best examples; it is a pet hate of ours as well. However, it is hard to get a grasp of exactly how many genes are involved in breast cancer and even harder to know how many are not. We have therefore decided to use gene annotation enrichment to give a sense of enrichment in specific biological backgrounds. These are now included in Figures 2-5. For Breast cancer classification, the most significant category is “breast cancer”; for response to treatment in ovarian cancer the category “molecule binding” and in response to chemo for breast cancer it is “cilium morphogenesis”. The latter is directly linked to efficiency of treatment in multiple cancers and primarily breast cancer1 .

Regarding the IDUA gene mutation we ask for a slight indulgence as our collaborators at the Institute of Cancer of Montpellier believe this may be a major finding. This mutation came out as second best and is directly linked to the uptake of the chemotherapeutic molecule used in the study. We are unable to put a statistic on how likely it is that amongst the 2 𝑘-mers with a good score one played a role in the binding of the exact chemo agent, but we estimate it is pretty low.
This last point meshed into the reviewers final concern: “because these examples all point to findings that are well known in the cancer literature, it is also unclear at the moment what novel findings can be achieved by iMOKA”. The IDUA gene is known to be important in binding of the chemotherapeutic agent, the SNP has been identified in a separate study; the link between the SNP and the binding of the chemotherapeutic agent is unknown. Because no other software will simultaneously search for SNPs, DE and isoforms this kind of information is lost in traditional RNA sequencing analysis.*


Reviewer #3: Deepening in their research on the use of 𝑘-mers for genomics data analysis, Lorenzi and colleagues have developed and implemented a new computational method called iMOKA that improves previous algorithms in its computational efficiency and accuracy. In my opinion, iMOKA represents a major improvement in the way genomics data might be analyzed. As clearly presented in datasets 2 and 3, its power lies in the integrative use of the data. If SNVs, gene expression or isoform usage, iMOKA is able to identify the best predictors in different biological setups without the need to explore all the sequences generated in a NGS experiment but only those that seem to be the best candidates. Also, as in dataset 3, there might be different features of a gene that can be relevant to a given disease. iMOKA also helps identifying the potential mechanisms of action of given genomic features, beyond the use of different technologies and data analysis pipelines.

There are only a few clarifications on the methodological decisions made, that should be done prior manuscript publication:
Methods:
* How did you come up with RF=10^9?
*The rescale factor is arbitrary and is used to reduce technical problems linked with precision. The value itself was chosen empirically to have usable values*
* I think the idea of the entropy filter is really great, to remove 𝑘-mers irrelevant for the classification of the samples. However, it is not totally clear to me how you update the threshold. Isnt it dangerous that it is adaptive? Couldn't you be removing relevant kmers as you go further in the process?
The pseudocode is shown in the figure Algorithm 2, that was not included by mistake in the manuscript and we deeply apologize for this.
*The adaptive filter has a great margin of tolerance to reduce the false negative ( features removed but that would have passed the accuracy filter) at the expense of the computational time. It’s not a standalone filter and is used to reduce the number of 𝑘-mers processed by the machine learning step. We have now included a supplementary figure (Figure S1) that shows this behaviour: In the 3 biggest datasets, none of the 𝑘-mers that would have been removed by the entropy filter would also have passed the accuracy filter, meanwhile in DLBCL, whose dimension is much smaller, the entropy filter threshold remains close to zero with only 4 𝑘-mers filtered incorrectly out of the 70497 𝑘-mers that passed the accuracy filter. The accuracy of those four 𝑘-mers, furthermore, is close to 65%, the value of the accuracy filter threshold used in that dataset.*

* Once more, there is not a convincing explanation of how to set the threshold for the overlapping of the 𝑘-mers in the graph
*The shift parameter is usually set to 1, so the 𝑘-mers need to overlap of k-1 nucleotides to form a link. In case of low coverage or high error rate, the resulting graphs can be fragmented in subgraphs and missing some edges. In those cases, we advise users to increase the threshold. We now include this advice in the method paragraph “𝑘-mer graph generation”*

* Graph mapping and annotation:
o alternative splice sites are identified when a 𝑘-mer graph is split across exons. How efficient is that? How do you do it? Coordinates comparison?
*The alternative splices sites are identified when there are two aligning sequences, derived from graphs, that present a split having one position in common and the other doesn’t. It’s possible to see an example of this in the supplementary figure 2 or opening the supplementary data 1 with the GUI ( File explorer -> 𝑘-mer list -> open the JSON file TCGA_BC_aggregated.json -> go to the 𝑘-mer list view and search the gene MYO6). Only one 𝑘-mer overlapping this event is kept, but you can see in the “Sequences” track that the exon in position chr6:75,898,373-75,898,411 is present in two sequences ( one in the + and one in the - strand ) , and absent in the other two. It’s efficient if the event of alternative splice is a marker useful to classify the groups, otherwise usually the most abundant isoform is detected.*

o differential expression (DE) is identified if 50% (set by parameter d) of an annotated transcript is covered by the 𝑘-mer graphs. Why 50%? Could you please clarify that?

*For DE it is essential to strike a balance between measuring a sufficiently large portion of the gene to overcome bias due to structural isoforms, SNP or indels and on the other hand reducing the required coverage of a gene to account for shorter transcripts or regions that are difficult to sequence. We propose 50% as the threshold through experience, but this can be different according to the type of data.
For example in the new mixed-gender dataset, DLBCL, the RNA-sequencing is targeted. This type of data produces specific fragments overlapping portions of transcripts and the only information that can be estimated is the abundance of the relative genes or transcripts, so the user can set the -d parameter to 1% or consider anyways all the 𝑘-mers produced as indicators of DE.
This threshold won’t add or remove any 𝑘-mers, but it will switch the event type from “gene” to “DE”. We included a clarification in the main text:
“Since regions with sequence variations not associated with the classes generate holes in the graphs reducing the portion of the transcripts that generate useful 𝑘-mers, a higher threshold might result in classifying DE event as general “gene” event, that is the best 𝑘-mer in a gene.“*


o Unmapped or multimapped events. How many of these do you get in average?
*The unampped or multimapped sequences depend strongly on the aligner used. The percentage of unmapped sequences is relatively high: 1,5%, 2,5% and 7,8% for BEAUTY, TCGA_BC and TCGA_OV respectively. Furthermore, since they cannot be aggregated by gene, all of the unmapped sequences are kept in the final result. We included a correlation parameter (-c in the CLI ) to aggregate the unmapped 𝑘-mers, but it is set to 1 ( disabled) in the results sent in order to avoid the elimination of potential interesting results.*

Don't you consider a probabilistic approach (EM like) to identify the most likely genomic location that generated the read?
*To be honest, we hadn’t thought of this. A probabilistic approach is a very good idea and we’ll start implementing it.*


How do you interpret those that are not annotated?
*Good question. An example of unmapped 𝑘-mer with an high score can be found in the TCGA_BC experiment, where “AATATTTAGTTTTTTTTTTTTTTTTTTTTAT” has a high rank with iMOKA. This sequence maps with BLAT (on the ESR1 gene) but does not map with GMAP. We believe that the stretch of ‘T’s may have prevented GMAP from mapping. Other factors that play into non-mappability from our experience are unannotated splice junctions and deletions or insertions.*

Results:
* Figure 2C. Would be nice to compare against plain gene quantification, probably these genes are very predictive anyway (i.e. ESR1)
*Yes indeed, we included the boxplot of the Gene counts of the same genes shown in figure 2C in the supplementary figure 3. They show the same behaviour as the associated 𝑘-mers*

* The OV cancer example is really nice. I agree that the gain might be due to the fact that the most discriminative 𝑘-mers are point mutations. Also the third example is really nice and shows the true potential of iMOKA
*Thanks, we can’t stress enough the amount of information that is lost in most RNA-seq experiments due to the analysis that is searching for only one specific feature (gene abundance, splicing...).*

**Second round of review**

**Reviewer 1**

Yes, the authors have satisfactorily responded to my previous review

**Reviewer 2**

All my previous concerns are well addressed.

**Reviewer 3**

Some letters/numbers/symbols are missing probably due to wrong pdf conversion in pages 14 and 17.
